# Supplementary material for: Photochemical permutation of meta-substituted phenols
Source: Nat Commun. 2025 Aug 13;16:7502. doi: 10.1038/s41467-025-62549-5 (PMC12350626; doi:10.1038/s41467-025-62549-5)
Supplement: Supplementary file 1 — Supplementary Information [file 41467_2025_62549_MOESM1_ESM.pdf]

Supplementary Information

Photochemical Permutation of *meta*-Substituted Phenols

Maialen Alonso,[#1] Giovanni Lonardi,[#1] Enrique M. Arpa,[1] Baptiste Roure,[1,2] Alessandro Ruffoni,*[3] and Daniele Leonori*[1]

[1] *Institute of Organic Chemistry, RWTH-Aachen University, Landoltweg 1, 52056, Aachen, Germany;*

[2] *Department of Chemistry, University of Manchester, Oxford Road, Manchester M13 9PL, UK;*

[3] *Otto Diels – Institute of Organic Chemistry, Christian Albrecht Universitat zu Kiel, Otto-Hahn-Platz 4, 24188 Kiel, Germany.*

[#] These authors contributed equally to this work.

* aruffoni@oc.uni-kiel.de and daniele.leonori@rwth-aachen.de

Table of content :

1 General Experimental Details 3

2 General Procedures 4

3 Starting Material Synthesis 6

4 Reaction Optimization 10

4.1 General Procedure for the Reaction Optimization of 3,5-dimethylphenol (1_1) 10

4.2 General Procedure for the Reaction Optimization of *m*-Cresol (2_2) 20

4.3 General Procedure for the Reaction Optimization of 3-phenylphenol (3_1) 22

4.4 General Procedure for the Reaction Optimization of 3,5-dimethylanisole (23_1) 24

4.5 Competition Experiments 26

5 Pictures of Reaction Set-up 27

6 UV-Vis Spectra 28

7 Substrate Scope 29

7.1 Additional Substrates 41

8 Computational Details 43

9 Computational Studies 45

9.1 Summary 50

10 Mechanism for Dimethyl Phenols (1_1-1_6) 51

11 Unsuccessful Substrates 55

12 NMR Spectra 56

13 Supplementary References 61

1 General Experimental Details

All required fine chemicals were used directly without purification unless stated otherwise. All air and moisture sensitive reactions were carried out under Argon atmosphere using standard Schlenk manifold technique. All solvents were bought from Acros as 99.8% purity and degassed by Ar bubbling. ^{1}H and ^{13}C Nuclear Magnetic Resonance (NMR) spectra were acquired at various field strengths as indicated and were referenced to $CHCl_3$ (7.27 and 77.16 ppm for ^{1}H and ^{13}C respectively). ^{1}H NMR coupling constants are reported in Hertz and refer to apparent multiplicities and not true coupling constants. Data are reported as follows: chemical shift, integration, multiplicity (s = singlet, br s = broad singlet, d = doublet, t = triplet, q = quartet, p = pentet, sx = sextet, hp = heptet, m = multiplet, dd = doublet of doublets, etc.). ^{19}F NMR spectra were recorded and reported unreferenced. High-resolution mass spectra were obtained using a JEOL JMS-700 spectrometer or a Fissions VG Trio 2000 quadrupole mass spectrometer. Spectra were obtained using electron impact ionization (EI) and chemical ionization (CI) techniques, or positive electrospray (ES). Analytical TLC: aluminum backed plates pre-coated (0.25 mm) with Merck Silica Gel 60 F254. Compounds were visualized by exposure to UV-light or by dipping the plates in permanganate ($KMnO_4$) or followed by heating. Flash column chromatography was performed using Merck Silica Gel 60 (40–63 µm). Absorption and emission spectra were obtained using an Horiba Duetta spectrometer and 1 mm High Precision Cell made of quartz from Hellma Analytics. All mixed solvent eluents are reported as v/v solutions. The LEDs used are Kessil PR 160 530, 440, 427, 390 and 370 nm. Reactions irradiated at 254, 310 and 350 nm were carried out in a Photochemical Multirays Reactor from HeliosQuartz equipped with the corresponding lamps. All the reactions were conducted in CEM 10 mL glass microwave tubes.

2 General Procedures

General Procedure for the Suzuki Cross Coupling – GP1

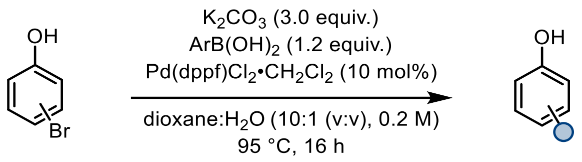

A 15 mL pressure vessel equipped with a stirring bar was charged with the boronic acid (1.2 equiv.), the bromo phenol (if solid; 1.0 equiv.), $Pd(dppf)Cl_2 \cdot CH_2Cl_2$ (10 mol%) and K_2CO_3 (3.0 equiv.). The pressure vessel was evacuated and refilled with N_2 (x 3). Dioxane and H_2O (10:1, 0.2 M) and the bromo phenol (if liquid; 1.0 equiv.) were added. The mixture was stirred for 16 h at 95 °C. After completion of the reaction as monitored by TLC, the mixture was diluted with EtOAc and $NaHCO_3$(sat). The layers were separated, and the aqueous layer was extracted with EtOAc. The combined organic layers were washed with brine (x 2), dried ($MgSO_4$) and filtered. The solvent was evaporated, and the residue was purified by column chromatography on silica gel to give the desired product.

General procedure for the Robinson annulation – GP2

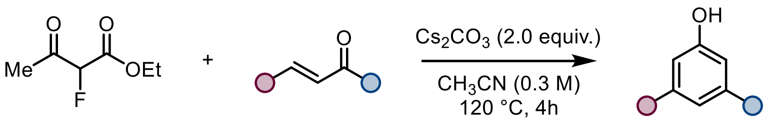

A round bottom flask equipped with a stirring bar was charged with ethyl 2-fluoro-3-oxobutanoate (1.1 equiv.), chalcone (1.0 equiv.), Cs_2CO_3 (2.0 mmol). The flask was evacuated and refilled with Ar (x 3) and CH_3CN (0.3 M) was added. The mixture was stirred at 120 °C for 4 h. After completion of the reaction as monitored by TLC, the pH of reaction mixture was adjusted to 4 with HCl (1 M) and CH_2Cl_2 was added. The layers were separated, and the aqueous layer was extracted with CH_2Cl_2 (x 2). The combined organic layers were washed with brine (x 2), dried ($MgSO_4$) and filtered. The solvent was evaporated, and the residue was purified by column chromatography on silica gel to give the desired product.

General procedure for reduction – GP3

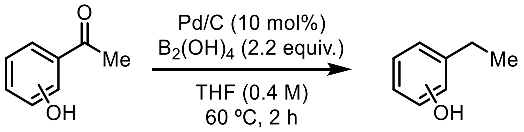

A round bottom flask equipped with a stirring bar was charged with 10% Pd/C (0.03 equiv), the corresponding hydroxyacetophenone (1.0 equiv.) and $B_2(OH)_4$ (2.2 equiv.). The flask was

evacuated and refilled with Ar (x 3) and THF (0.4 M) was added. The mixture was stirred at 60 °C for 2 h. After completion of the reaction as monitored by TLC, the crude mixture was filtered through a celite plug eluting with CH_2Cl_2/MeOH (95:5). The solvent was evaporated, and the residue was purified by column chromatography on silica gel to give the desired product.

3 Starting Material Synthesis

4'-Methyl-[1,1'-biphenyl]-3-ol (4_1)

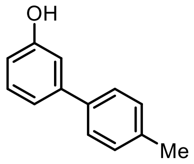

Following **GP1**, 3-bromophenol (520 mg, 3 mmol, 1.0 equiv.) and 4-methylphenylboronic acid (490 mg, 3.6 mmol, 1.2 equiv.) gave **4₁** (398 mg, 72%) as a solid. ^{1}H NMR ($CDCl_3$, 600 MHz) δ 7.48 (2H, d, *J* = 8.3 Hz), 7.30 (1H, t, *J* = 7.9 Hz), 7.25 (2H, d, *J* = 8.3 Hz), 7.16 (1H, ddd, *J* = 7.7, 1.7, 0.9 Hz), 7.05 (1H, dd, *J* = 2.6, 1.7 Hz), 6.80 (1H, ddd, *J* = 8.0, 2.6, 1.0 Hz), 4.90 (1H, s), 2.40 (3H, s); ^{13}C NMR ($CDCl_3$, 151 MHz) δ 155.9, 143.1, 138.0, 137.4, 130.1, 129.6, 127.1, 119.7, 114.0 (2C), 21.2. Data in accordance with the literature.[1]

4'-Fluoro-[1,1'-biphenyl]-3-ol (5_1)

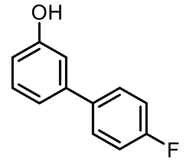

Following **GP1**, 3-bromophenol (400 mg, 2.3 mmol, 1.0 equiv.) and 4-fluorophenylboronic acid (388 mg, 2.8 mmol, 1.2 equiv.) gave **5_1** (650 mg, 79%) as a solid. ^{1}H NMR (400 MHz, $CDCl_3$) δ 7.56 – 7.49 (2H, m), 7.30 (1H, t, *J* = 7.9 Hz), 7.16 – 7.08 (3H, m), 7.01 (1H, dd, *J* = 2.6, 1.7 Hz), 6.81 (1H, ddd, *J* = 8.0, 2.6, 1.0 Hz), 4.81 (1H, s); ^{13}C NMR ($CDCl_3$, 151 MHz) δ 163.5, 161.9, 156.0, 142.2, 137.0, 130.2, 128.8 (d, *J* = 7.7 Hz), 119.8, 115.8 (d, *J* = 21.5 Hz), 114.2 (d, *J* = 28.2 Hz). ^{19}F NMR ($CDCl_3$, 564 MHz) δ -115.45. Data in accordance with the literature.[2]

3-Ethyl-5-methylphenol (6_1)

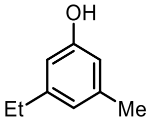

Following **GP2**, 4-hexen-3-one (491 mg, 5 mmol, 1.0 equiv.) and ethyl-2-floroacetoacetate (889 mg, 6 mmol, 1.2 equiv.) gave **6_1** (571 mg, 84%) as a solid. R_f 0.31 [pentane:EtOAc (9:1)]. ^{1}H NMR ($CDCl_3$, 600 MHz) δ 6.61 (1H, s), 6.50 (1H, s), 6.49 (1H, s), 4.84 (1H, br s), 2.57 (2H, q, *J* = 7.6 Hz), 2.28 (3H, s), 1.21 (3H, t, *J* = 7.6 Hz); ^{13}C NMR ($CDCl_3$, 151 MHz) δ 155.6,

146.2, 139.7, 121.4, 113.4, 111.9, 28.8, 21.5, 15.6. HRMS (EI): found M^+ 136.0886, $C_9H_{12}O$ requires 136.0888.

5-Methyl-[1,1'-biphenyl]-3-ol (7_l)

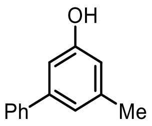

Following **GP2**, 4-phenyl-3-buten-2-on (731 mg, 5 mmol, 1.0 equiv.) and ethyl 2-fluoroacetoacetate (889 mg, 6 mmol, 1.2 equiv.) gave **7_l** (663 mg, 72%) as a solid. ^{1}H NMR ($CDCl_3$, 600 MHz) δ 7.56 (2H, dd, J = 8.3, 1.2 Hz), 7.44 – 7.40 (2H, m), 7.38 – 7.29 (1H, m), 7.00 (1H, t, J = 1.6 Hz), 6.87 (1H, t, J = 2.0 Hz), 6.65 (1H, t, J = 2.3 Hz), 4.75 (1H, s), 2.37 (3H, s); ^{13}C NMR ($CDCl_3$, 151 MHz) δ 155.9, 143.0, 141.0, 140.3, 128.8, 127.5, 127.3, 120.9, 115.1, 111.4, 21.6. Data in accordance with the literature.[3]

2-Ethyl-5-methylphenol (10_l)

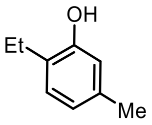

Following **GP3**, 2'-hydroxy-4'-methylacetophenone (450 mg, 3 mmol, 1.0 equiv.) gave **10_l** (155 mg, 38%) as an oil. ^{1}H NMR ($CDCl_3$, 400 MHz) δ 7.04 (1H, d, J = 7.6 Hz), 6.72 (1H, d, J = 7.8 Hz), 6.61 (1H, s), 4.57 (1H, s), 2.62 (2H, q, J = 7.5 Hz), 2.30 (3H, s), 1.24 (3H, t, J = 7.6 Hz); ^{13}C NMR ($CDCl_3$, 101 MHz) δ 153.1, 137.0, 129.1, 127.5, 123.6, 116.1, 22.6, 21.0, 14.4. Data in accordance with the literature.[4]

4-Methyl-[1,1'-biphenyl]-2-ol (11_l)

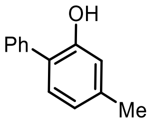

Following **GP1**, 6-bromo-3-methylphenol (748 mg, 4 mmol, 1.0 equiv.) and $PhB(OH)_2$ (536 mg, 4.4 mmol, 1.1 equiv.) gave **11_l** (397 mg, 76%) as a solid. ^{1}H NMR ($CDCl_3$, 600 MHz) δ 7.51 – 7.44 (4H, m), 7.42 – 7.36 (1H, m), 7.15 (1H, d, J = 8.0 Hz), 6.85 – 6.81 (2H, m), 5.16 (1H, s), 2.37 (3H, s); ^{13}C NMR ($CDCl_3$, 151 MHz) δ 152.6, 139.2, 137.6, 130.2, 129.2, 129.0, 127.4, 125.4, 121.6, 116.5, 21.2. Data in accordance with the literature.[5]

4-Ethyl-3-methylphenol (16_l)

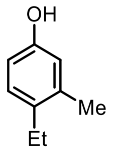

Following **GP3**, 4'-hydroxy-2'-methylacetophenone (451 mg, 3 mmol, 1.0 equiv.) gave **16_l** (266 mg, 65%) as an oil. R_f 0.50 [pentane:EtOAc (9:1)]. ^{1}H NMR ($CDCl_3$, 600 MHz) δ 7.01 (1H, d, J = 8.1 Hz), 6.65 – 6.59 (2H, m), 4.46 (1H, s), 2.55 (2H, q, J = 7.5 Hz), 2.26 (3H, s), 1.17 (3H, t, J = 7.6 Hz); ^{13}C NMR ($CDCl_3$, 101 MHz) δ 153.4, 137.4, 134.9, 129.1, 117.0, 112.7, 25.5, 19.4, 14.8. HRMS (EI): found M^+ 136.0882, $C_9H_{12}O$ requires 136.0888.

2-Methyl-[1,1'-biphenyl]-4-ol (17_l)

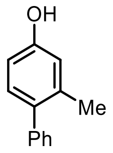

Following **GP1**, 4-bromo-3-methylphenol (748 mg, 4 mmol, 1.0 equiv.) and $PhB(OH)_2$ (536 mg, 4.4 mmol, 1.1 equiv.) gave **17_l** (444 mg, 85%) as a solid. ^{1}H NMR ($CDCl_3$, 400 MHz) δ 7.37 (2H, t, J = 7.3 Hz), 7.31 – 7.27 (3H, m), 7.08 (1H, d, J = 8.2 Hz), 6.76 (1H, d, J = 2.7 Hz), 6.72 (1H, dd, J = 8.2, 2.6 Hz), 5.96 (1H, s), 2.21 (3H, s); ^{13}C NMR ($CDCl_3$, 151 MHz) δ 154.7, 141.7, 137.2, 135.0, 131.2, 129.5, 128.2, 126.6, 117.1, 112.8, 20.7. Data in accordance with the literature.[6]

6-Methyl-[1,1'-biphenyl]-2-ol (18_l)

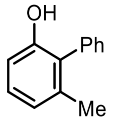

Following **GP1**, 2-bromo-3-methylphenol (748 mg, 4 mmol, 1.0 equiv.) and $PhB(OH)_2$ (536 mg, 4.4 mmol, 1.1 equiv.) gave **18_l** (366 mg, 70%) as a solid. ^{1}H NMR ($CDCl_3$, 400 MHz) δ 7.50 (2H, t, J = 7.3 Hz), 7.41 (1H, t, J = 7.4 Hz), 7.31 – 7.27 (2H, m), 7.15 (1H, t, J = 7.9 Hz), 6.84 (2H, d, J = 6.8 Hz), 4.84 (1H, s), 2.06 (3H, s); ^{13}C NMR ($CDCl_3$, 151 MHz) δ 153.0, 137.3, 135.4, 130.4, 129.5, 128.6, 128.3, 128.2, 122.1, 112.8, 20.5. Data in accordance with the literature.[7]

2,6-Dimethyl-[1,1'-biphenyl]-4-ol (20_1)

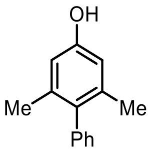

Following **GP1**, 3-bromo-3,5-dimethylphenol (804 mg, 4 mmol, 1.0 equiv.) and $PhB(OH)_2$ (536 mg, 4.4 mmol, 1.1 equiv.) gave **20_1** (603 mg, 76%) as a solid. ^{1}H NMR ($CDCl_3$, 600 MHz) δ 7.40 (2H, t, J = 7.4 Hz), 7.31 (1H, t, J = 7.4 Hz), 7.12 (2H, dd, J = 8.2, 1.4 Hz), 6.59 (2H, s), 5.46 (1H, s), 1.98 (6H, s); ^{13}C NMR ($CDCl_3$, 101 MHz) δ 154.6, 141.0, 137.8, 134.6, 129.8, 128.4, 126.6, 114.1, 21.0. Data in accordance with the literature.[6]

4 Reaction Optimization

A disubstituted phenol (3,5-dimethylphenol) was selected for the full optimization of the reaction parameters in order to have a more complete analysis of the reaction.

4.1 General Procedure for the Reaction Optimization of 3,5-dimethylphenol ($\mathbf{1_1}$)

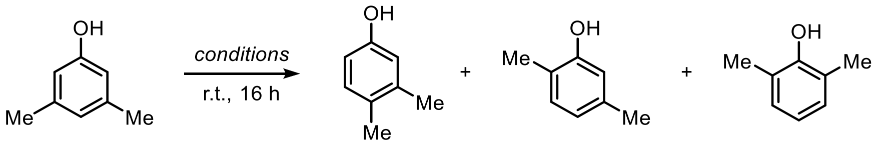

$\mathbf{1_1}$ $\mathbf{1_2}$ $\mathbf{1_3}$ $\mathbf{1_4}$

In an Argon filled glove box, a dry tube equipped with a stirring bar was charged with 3,5-dimethylphenol **($\mathbf{1_1}$)** (16 mg, 0.1 mmol, 1.0 equiv.) followed by the corresponding acid (if solid, 0.5 equiv.). The corresponding anhydrous and degassed solvent and the acid (if liquid, 0.5 equiv.) were added and the tube was capped with a Supelco aluminium crimp seal with septum (PTFE/butyl). The tube was placed into a Helios photoreactor equipped with the corresponding lamps and a fan (for reactions at 254 and 310 nm) or under a Kessil lamp with a distance from the lamp to the bottom of the vial of 4 cm (for reactions at 370 nm, 390 nm and 427 nm). The lamps and the fan were switched on and the mixture was stirred under irradiation. The mixture was diluted with H_2O (2 mL). 1,3- Dinitrobenzene (17 mg, 0.1 mmol, 1.0 equiv.) was added and the layers were separated. The aqueous layer was extracted with CH_2Cl_2 (2 mL x 2) and the combined organic layers were dried ($MgSO_4$), filtered and evaporated. The crude was dissolved in $CDCl_3$ (0.6 mL) and analysed by ^{1}H NMR spectroscopy to obtain the reaction quantitative NMR yield.

Table S1. Screening of wavelengths.

entry	*hv* (nm)	time (h)	1_2 (%)	1_3 (%)	1_4 (%)	1_1 (%)
**1**	254	16	-	-	-	-
**2**	310	8	47	11	-	23
**3**	310	16	**63**	11	-	11
**4**	370	16	-	-	80	-
**5**	390	2	-	-	**99**	-
**6**	390	16	-	-	90	-
**7**	427	2	-	19	20	60
**8**	427	16	-	-	84	-

Considering the different selectivity observed by changing the wavelength, the rest of the reaction parameters were optimized both at 310 nm and 390 nm.

Optimization of the reaction parameters at 310 nm

Table S2. Screening of solvents using $AlBr_3$ (0.5 equiv.) as the acid.

entry	solvent (0.1 M)	time (h)	1_2 (%)	1_3 (%)	1_4 (%)	1_1 (%)
**1**	CH_2Cl_2	8	**47**	11	-	23
**2**	$CHCl_3$	8	-	-	-	13
**3**	MeCN	8	-	-	-	92
**4**	THF	8	-	-	-	99
**5**	Dioxane	8	-	-	-	91
**6**	Et_2O	8	-	-	-	95
**7**	DCE	8	6	-	-	48
**8**	EtOAc	8	-	-	-	93
**9**	Acetone	8	-	-	-	99
**10**	HFIP	8	-	-	-	90

Table S3. Screening of concentration using $AlBr_3$ (0.5 equiv.) as the acid.

entry	time (h)	concentration (M)	1_2 (%)	1_3 (%)	1_4 (%)	1_1 (%)
**1**	16	0.2	30	14	-	57
**2**	16	0.1	**63**	11	-	12
**3**	16	0.05	60	23	-	-
**4**	16	0.025	47	23	-	-

Table S4. Screening of reaction time in CH_2Cl_2 (0.1 M) using $AlBr_3$ (0.5 equiv.) as the acid.

entry	time (h)	1_2 (%)	1_3 (%)	1_4 (%)	1_1 (%)
**1**	1	5	-	-	79
**2**	2	14	<5	-	70
**3**	6	34	7	-	37
**4**	8	47	11	-	23
**5**	16	**63**	11	-	12
**6**	24	-	-	-	-

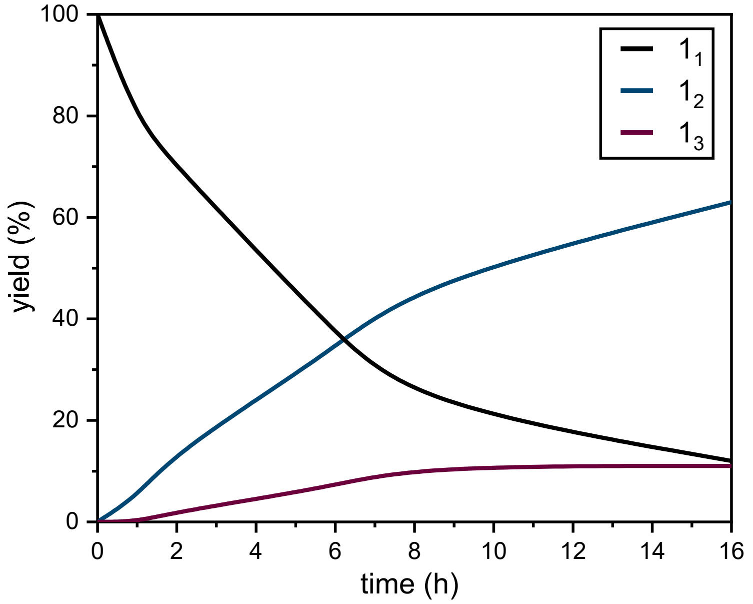

Figure S1. Reaction over time at 310 nm.

Table S5. Screening of acids in CH_2Cl_2 (0.1 M).

**entry**	**acid (0.5 equiv.)**	**time (h)**	**1_2 (%)**	**1_3 (%)**	**1_4 (%)**	**1_1 (%)**
**1**	$AlBr_3$	8	**47**	11	-	23
**2**	$B(C_6F_5)_3$	8	-	-	-	37
**3**	$Fe(OTf)_3$	8	-	-	-	40
**4**	Ag(OTf)	8	-	-	-	87
**5**	$Cu(OTf)_2$	8	-	-	-	93
**6**	$Zn(OTf)_2$	8	-	-	-	90
**7**	$Sc(OTf)_3$	8	-	-	-	93
**8**	$Ce(OTf)_3$	8	-	-	-	93
**9**	$Al(OTf)_3$	8	-	-	-	91
**10**	$InBr_3$	8	-	-	-	94
**11**	$FeCl_3$	8	-	-	-	60
**12**	$ZnBr_2$	8	-	-	-	99
**13**	CuBr	8	-	-	-	97
**14**	$AlCl_3$	8	40	10	-	<5
**15**	$CeCl_3$	8	-	-	-	40
**16**	BBr_3	8	-	-	-	90
**17**	$BF_3{\cdot}OEt_2$	8	-	-	-	88
**18**	TfOH	8	34	9	-	34

Table S6. Screening of $AlBr_3$ loading in CH_2Cl_2 (0.1 M).

entry	time (h)	acid (equiv.)	1_2 (%)	1_3 (%)	1_4 (%)	1_1 (%)
**1**	8	-	-	-	-	94
**2**	8	0.1	6	22	-	63
**3**	8	0.3	33	9	-	54
**4**	8	0.5	**47**	12	-	23
**5**	8	0.7	27	17	-	63
**6**	8	1	12	17	-	64
**7**	8	1.2	27	7	-	57
**8**	8	1.5	15	7	-	62
**9**	8	2	7	5	-	61
**10**	8	5	5	<5	-	60

Optimization of the reaction parameters at 390 nm

Table S7. Screening of solvents using $AlBr_3$ (0.5 equiv.) as the acid.

entry	solvent (0.1 M)	time (h)	1_2 (%)	1_3 (%)	1_4 (%)	1_1 (%)
**1**	CH_2Cl_2	2	-	-	**99**	-
**2**	$CHCl_3$	2	-	-	80	-
**3**	MeCN	2	-	-	-	99
**4**	THF	2	-	-	-	99
**5**	Dioxane	2	-	-	-	95
**6**	Et_2O	2	-	-	-	95
**7**	DCE	2	-	-	84	-
**8**	EtOAc	2	-	-	-	99
**9**	Acetone	2	-	-	-	99
**10**	HFIP	2	-	-	-	98
**11**	Toluene	2	-	-	88	-

Table S8. Screening of concentration using $AlBr_3$ (0.5 equiv.) as the acid.

entry	time (h)	concentration (M)	1_2 (%)	1_3 (%)	1_4 (%)	1_1 (%)
**1**	2	0.2	-	-	98	-
**2**	2	0.1	-	-	**99**	**-**
**3**	2	0.05	-	-	97	-
**4**	2	0.025	-	-	98	-

Table S9. Screening of reaction time in CH_2Cl_2 (0.1 M) using $AlBr_3$ (0.5 equiv.) as the acid.

entry	time (h)	1_2 (%)	1_3 (%)	1_4 (%)	1_1 (%)
**1**	15 min		46		24
**2**	30 min		68		
**3**	1	-	22	63	-
**4**	2	-	-	**99**	-
**5**	6	-	-	89	-
**6**	8	-	-	87	-
**7**	16	-	-	84	-
**8**	24	-	-	74	-

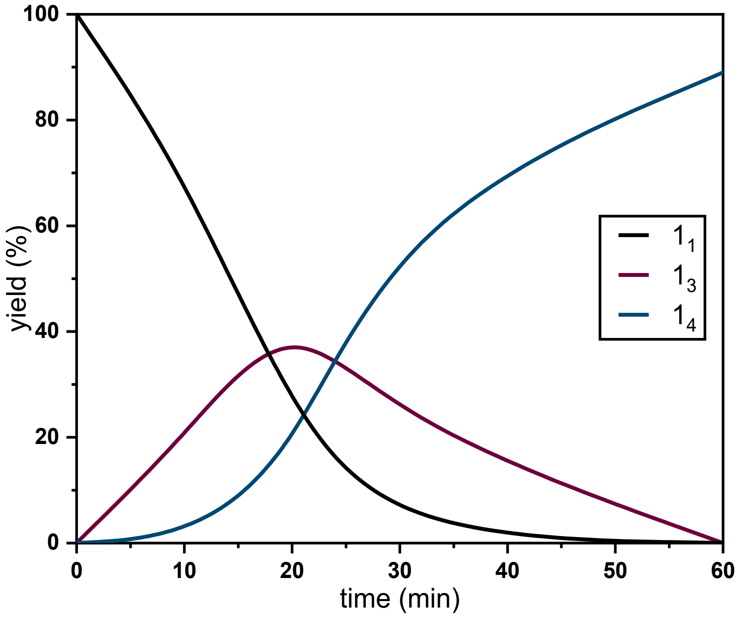

Figure S2. Reaction over time at 390 nm.

Table S10. Screening of acids in CH_2Cl_2 (0.1 M).

entry	acid (0.5 equiv.)	time (h)	1_2 (%)	1_3 (%)	1_4 (%)	1_1 (%)
**1**	$AlBr_3$	2	-	-	**99**	-
**2**	$B(C_6F_5)_3$	2	-	**46**	-	-
**3**	$Fe(OTf)_3$	2	-	-	-	88
**4**	Ag(OTf)	2	-	-	-	-
**5**	$Cu(OTf)_2$	2	-	-	-	99
**6**	$Zn(OTf)_2$	2	-	-	-	99
**7**	$Sc(OTf)_3$	2	-	-	-	99
**8**	$Ce(OTf)_3$	2	-	-	-	98
**9**	$Al(OTf)_3$	2	-	-	-	98
**10**	$InBr_3$	2	-	-	-	99
**11**	$FeCl_3$	2	-	-	-	80
**12**	$ZnBr_2$	2	-	-	-	98
**13**	CuBr	2	-	-	-	99
**14**	$AlCl_3$	2	-	-	92	-
**15**	$CeCl_3$	2	-	-	-	98
**16**	BBr_3	2	-	-	-	90
**17**	$BF_3.OEt_2$	2	-	-	-	95
**18**	TfOH	2	-	24	26	-

Table S11. Screening of $AlBr_3$ loading in CH_2Cl_2 (0.1 M).

entry	time (h)	acid (equiv.)	1_2 (%)	1_3 (%)	1_4 (%)	1_1 (%)
**1**	2	-	-	-	-	98
**2**	2	0.1	-	-	99	-
**3**	2	0.3	-	-	99	-
**4**	2	0.5	-	-	**99**	-
**5**	2	0.7	-	-	99	-
**6**	2	1	-	-	99	-
**7**	2	1.2	-	-	93	-
**8**	2	1.5	-	-	27	-
**9**	2	2	-	-	-	-*
**10**	2	5	-	-	-	-*

*: decomposition of the starting material.

Tests on the Photostability of all Dimethylphenol Isomers at 310 and 390 nm

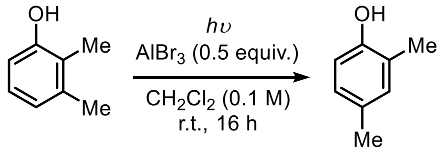

1_6 1_5

Table S12. Screening of wavelengths.

entry	*hυ* (nm)	1_5 (%)	1_6 (%)
**1**	310	**30**	-
**2**	390	-	35

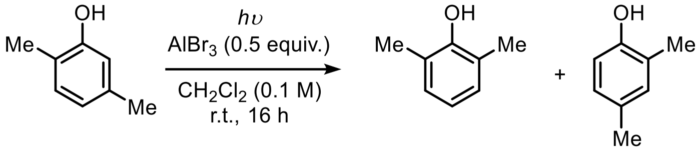

1_3 1_4 1_5

Table S13. Screening of wavelengths.

entry	*hυ* (nm)	1_4 (%)	1_5 (%)	1_3 (%)
**1**	310	16	**50**	24
**2**	390	**99**	-	-

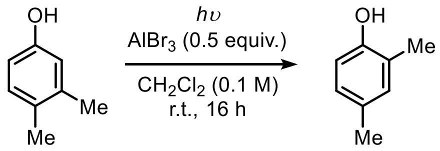

1_2 1_5

Table S14. Screening of wavelengths.

entry	*hυ* (nm)	1_5 (%)	1_2 (%)
**1**	310	16	40
**2**	390	**63**	-

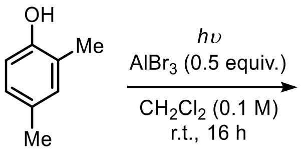

Table S15. Screening of wavelengths.

entry	*hυ* (nm)	1_5 (%)
**1**	310	50
**2**	390	92

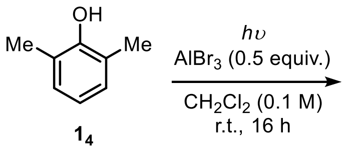

Table S16. Screening of wavelengths.

entry	*hυ* (nm)	1_4 (%)
**1**	310	77
**2**	390	99

4.2 General Procedure for the Reaction Optimization of *m*-Cresol (2_2)

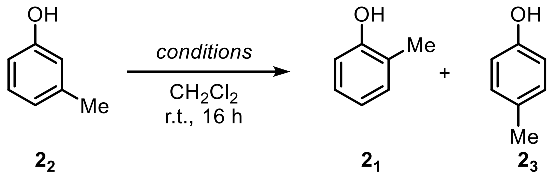

In an Argon filled glove box, a dry tube equipped with a stirring bar was charged with 3-phenylphenol **(2_2)** (10 mg, 0.1 mmol, 1.0 equiv.) and $AlBr_3$ (13 mg, 0.5 equiv.) followed by dry and degassed CH_2Cl_2. The tube was capped with a Supelco aluminium crimp seal with septum (PTFE/butyl) and placed into a Helios photoreactor equipped with 310 nm lamps and a fan or under a Kessil lamp with a distance from the lamp to the bottom of the vial of 4 cm (for reactions at 390 nm). The lamps and the fan were switched on and the mixture was stirred under irradiation for 16 h. The mixture was diluted with H_2O (2 mL). 1,3- Dinitrobenzene (17 mg, 0.1 mmol, 1.0 equiv.) was added and the layers were separated. The aqueous layer was extracted with CH_2Cl_2 (2 mL x 2) and the combined organic layers were dried ($MgSO_4$), filtered and evaporated. The crude was dissolved in $CDCl_3$ (0.6 mL) and analysed by ^1H NMR spectroscopy to obtain the reaction quantitative NMR yield.

Table S17. Screening of wavelength in CH_2Cl_2 (0.1 M).

entry	*hν* (nm)	2_1 (%)	2_3 (%)	2_2 (%)
**1**	310	**20**	-	53
**2**	390	**-**	**64**	15
**3**	*no light*	**-**	**-**	93

Optimization of the reaction at 310 nm

Table S18. Screening of acid, concentration and time in CH_2Cl_2 (0.1 M).

entry	acid (0.5 equiv.)	concentration (M)	time (h)	2_1 (%)	2_3 (%)	2_2 (%)
**1**	$AlBr_3$	0.1	16	20	-	52
**2**	TfOH	0.1	16	42	-	32
**3**	$AlBr_3$	0.05	16	**68**	-	8
**4**	$AlBr_3$	0.1	24	**34**	-	37
**5**	*no acid*	0.1	16	**-**	-	98

Optimization of the reaction at 390 nm

Table S19. Screening of acid, concentration and time in CH_2Cl_2 (0.1 M).

entry	acid (0.5 equiv.)	concentration (M)	time (h)	2_1 (%)	2_3 (%)	2_2 (%)
**1**	$AlBr_3$	0.1	16	-	**64**	15
**2**	TfOH	0.1	16	**4**	27	31
**3**	BCF	0.1	16	**-**	-	99
**4**	$AlBr_3$	0.05	16	14	63	
**5**	$AlBr_3$	0.1	24	23	63	-

Tests on the Photostability of all Methylphenol Isomers at 310 and 390 nm

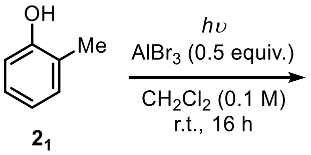

2_1

Table S12. Screening of wavelengths.

entry	*hν* (nm)	2_1 (%)
**1**	310	66
**2**	390	76

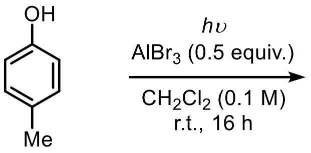

2_3

Table S12. Screening of wavelengths.

entry	*hν* (nm)	2_3 (%)
**1**	310	50
**2**	390	92

4.3 General Procedure for the Reaction Optimization of 3-phenylphenol (3_1)

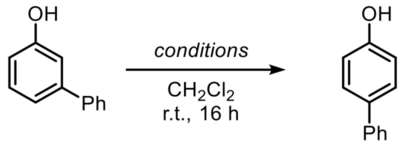

3_1 3_2

In an Argon filled glove box, a dry tube equipped with a stirring bar was charged with 3-phenylphenol **(3_1)** (17 mg, 0.1 mmol, 1.0 equiv.) and $AlBr_3$ (13 mg, 0.5 equiv.) followed by dry and degassed CH_2Cl_2. The tube was capped with a Supelco aluminium crimp seal with septum (PTFE/butyl) and placed into a Helios photoreactor equipped with 310 nm lamps and a fan or under a Kessil lamp with a distance from the lamp to the bottom of the vial of 4 cm (for reactions at 390 nm, 427 nm, 440 nm and 530 nm). The lamps and the fan were switched on and the mixture was stirred under irradiation for 16 h. The mixture was diluted with H_2O (2 mL). 1,3-Dinitrobenzene (17 mg, 0.1 mmol, 1.0 equiv.) was added and the layers were separated. The aqueous layer was extracted with CH_2Cl_2 (2 mL x 2) and the combined organic layers were dried ($MgSO_4$), filtered and evaporated. The crude was dissolved in $CDCl_3$ (0.6 mL) and analysed by ^{1}H NMR spectroscopy to obtain the reaction quantitative NMR yield.

Table S20. Screening of wavelength in CH_2Cl_2 (0.1 M).

**entry**	***hν* (nm)**	**3_2 (%)**	**3_1 (%)**
**1**	310	7	72
**2**	390	**22**	55
**3**	427	-	86
**4**	440	-	95
**5**	530	-	90

Table S21. Screening of concentration in CH_2Cl_2 at 390 nm.

**entry**	**concentration (M)**	**3_2 (%)**	**3_1 (%)**
**1**	0.1	22	55
**2**	0.05	**45**	33
**3**	0.025	32	25
**4***	0.05	21	38

*: reaction performed with 2.0 equiv. of TfOH instead of $AlBr_3$.

Table S22. Screening of time in CH_2Cl_2 (0.05 M) at 390 nm.

entry	time (h)	3_2 (%)	3_1 (%)
**1**	16 h	**45**	33
**2**	24 h	43	26
**3**	48 h	37	21

4.4 General Procedure for the Reaction Optimization of 3,5-dimethylanisole (23_1)

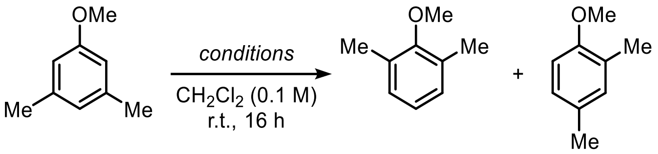

23_1 23_2 23_3

In an Argon filled glove box, a dry tube equipped with a stirring bar was charged with 3,5-dimethylanisole **(23_1)** (18 mg, 0.1 mmol, 1.0 equiv.) followed by dry and degassed CH_2Cl_2 (0.1 M) and the corresponding amount of triflic acid. The tube was capped with a Supelco aluminium crimp seal with septum (PTFE/butyl) and placed into a Helios photoreactor equipped with 310 mn lamps and a fan or under a 390 nm Kessil lamp with a distance from the lamp to the bottom of the vial of 4 cm. The lamps and the fan were switched on and the mixture was stirred under irradiation for 16 h. The mixture was diluted with H_2O (2 mL). 1,3-Dinitrobenzene (17 mg, 0.1 mmol, 1.0 equiv.) was added and the layers were separated. The aqueous layer was extracted with CH_2Cl_2 (2 mL x 2) and the combined organic layers were dried ($MgSO_4$), filtered and evaporated. The crude was dissolved in $CDCl_3$ (0.6 mL) and analysed by ^{1}H NMR spectroscopy to obtain the reaction quantitative NMR yield.

Optimization of the reaction at 310 nm

Table S23. Screening of TfOH loading in CH_2Cl_2 (0.1 M).

entry	TfOH (equiv.)	23_2 (%)	23_3 (%)	23_1 (%)
**1**	1.0 equiv. $AlBr_3$	-	-	88
**2**	0	-	-	92
**3**	0.5	-	-	59
**4**	1	-	20	50
**5**	2	-	**27**	44
**6**	5	-	19	60
**7**	10	-	30	45

Optimization of the reaction at 390 nm

Table S24. Screening of TfOH loading in CH_2Cl_2 (0.1 M).

entry	TfOH (equiv.)	23_2 (%)	23_3 (%)	23_1 (%)
**1**	1.0 equiv. $AlBr_3$	-	-	94
**2**	0	-	-	99
**3**	0.5	-	-	51
**4**	1	-	-	55
**5**	2	8	-	27
**6**	5	**25**	-	31
**7**	10	24	-	17

4.5 Competition Experiments.

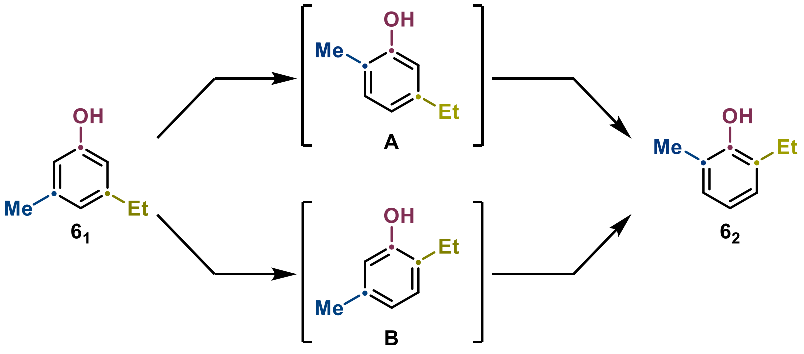

hν (nm)	additive	solvent	time	6_2 (%)	A (%)	B (%)	6_1 (%)
390	$AlBr_3$ (0.5 eq)	CH_2Cl_2 (0.1 M)	2 h	21	19	-	15
390	$AlBr_3$ (0.5 eq)	CH_2Cl_2 (0.1 M)	16 h	29	-	-	-

Under our optimized conditions (16 h) we only observe **6_2** as reported in the manuscript. However, reaction analysis after 2 h of irradiation revealed the formation of 19% of **A** and 21% of **6_2** and 15% of unreacted **6_1**. Crucially, we did not observe the formation of **B**. We believe this suggests that the Me group shift is faster than the Et group and this might be a result of sterics.

5 Pictures of Reaction Set-up

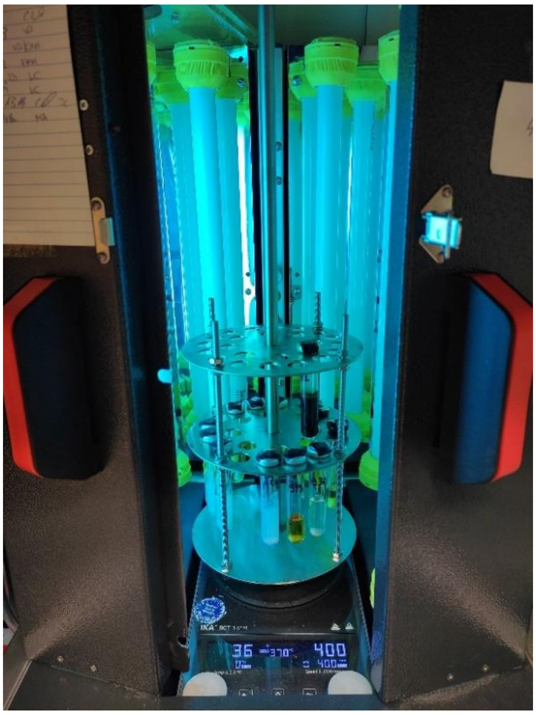

Figure S3. Set-up for 0.1 mmol scale reactions at 254, 310 and 350 nm.

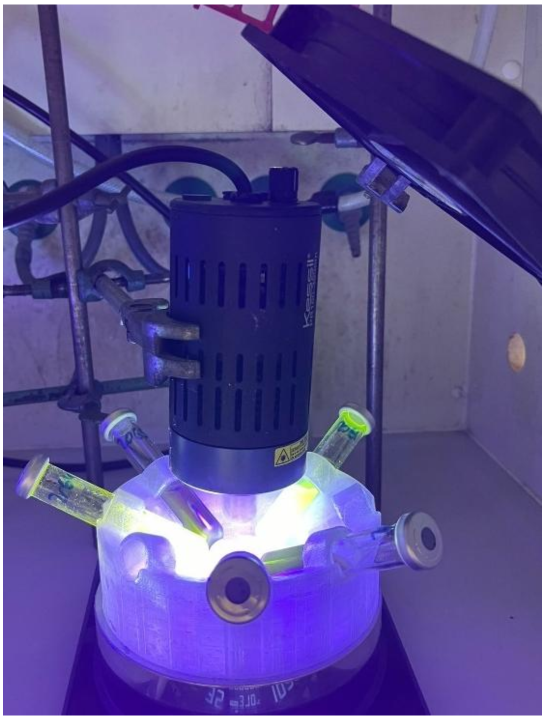

Figure S4. Set-up for 0.1 mmol scale reactions at 370, 390, 427, 440 and 530 nm.

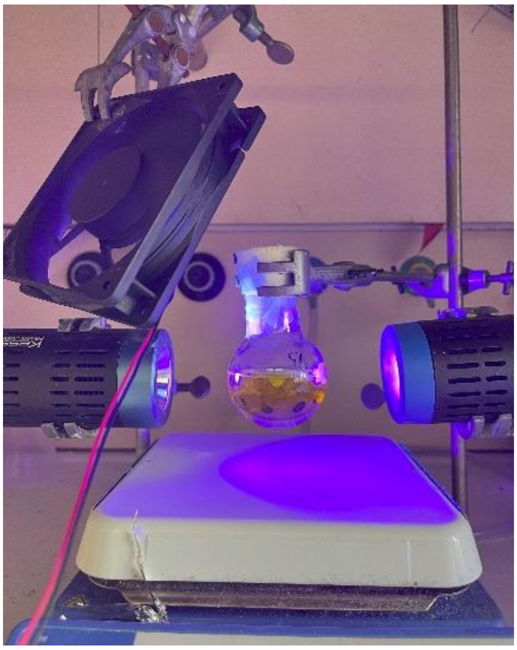

Figure S5. Set-up for gram-scale reactions at 390 nm.

6 UV-Vis Spectra

These analyses were not conducted using $AlBr_3$ because the high dilution required for good resolution leads to decomplexation. The UV-Vis spectra were recorded in pure TfOH in order to favour the protonated species in solution.

UV-Vis Spectra of 3,5-dimethylphenol (1$_1$)

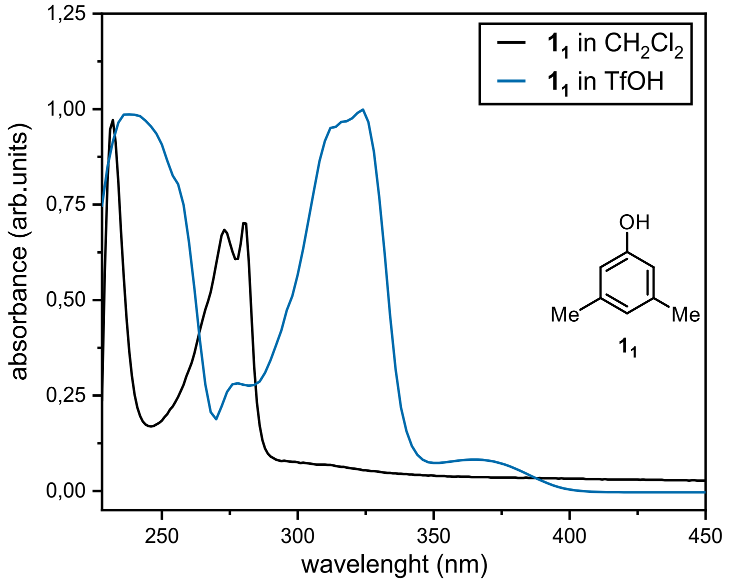

Figure S6. UV-Vis of 3,5-dimethylphenol (**1$_1$**)

UV-Vis Spectra of [1,1'-biphenyl]-3-ol (3$_1$)

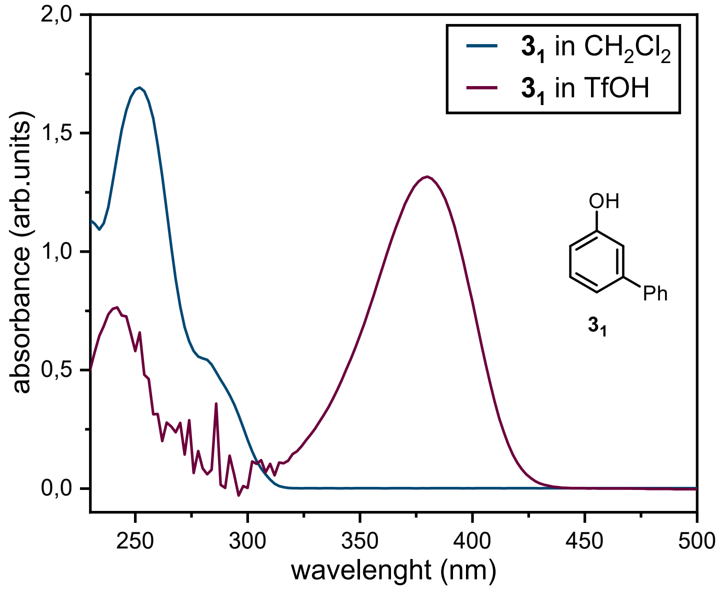

Figure S7. UV-Vis of [1,1'-biphenyl]-3-ol (**3$_1$**)

7 Substrate Scope

General Procedure for the Permutation of *meta*-Phenols – GP4

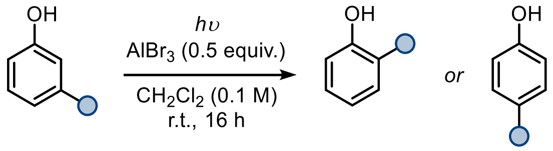

In an Argon filled glove box, a dry tube equipped with a stirring bar was charged with the corresponding phenol (0.1 mmol, 1.0 equiv.) followed by $AlBr_3$ (0.5 equiv.). Dry and degassed CH_2Cl_2 (0.1 M) was added and the tube was capped with a Supelco aluminium crimp seal with septum (PTFE/butyl). The tube was placed into a Helios photoreactor equipped with the 310 nm lamps and a fan (**GP4a**) or under a 390 nm Kessil lamp with a distance from the lamp to the bottom of the vial of 4 cm (**GP4b**). The lamps and the fan were switched on and the mixture was stirred under irradiation for 16 h. The photoreactor and the fan were switched off. The mixture was diluted with H_2O (2 mL). The organic layer was separated and the aqueous layer was extracted with CH_2Cl_2 (2 mL x 2) and the combined organic layers were dried (MgSO4), filtered and evaporated. The residue was purified by column chromatography on silica gel to give the desired product.

3,4-Dimethylphenol ($\mathbf{1_2}$)

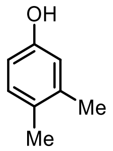

Following **GP4a**, $\mathbf{1_1}$ (13 mg, 0.1 mmol, 1.0 equiv.) gave $\mathbf{1_2}$ (63 %, 11 % $\mathbf{1_3}$, 12 % rsm) as a solid. ^{1}H NMR ($CDCl_3$, 400 MHz) δ 7.01 (1H, d, J = 8.1 Hz), 6.68 (1H, d, J = 2.8 Hz), 6.62 (1H, dd, J = 8.1, 2.8 Hz), 5.27 (1H, s), 2.23 (3H, s), 2.21 (3H, s); ^{13}C NMR ($CDCl_3$, 101 MHz) δ 153.5, 138.1, 130.6, 128.8, 116.8, 112.5, 19.9, 18.9. Data in accordance with the literature.[8]

2,5-Dimethylphenol ($\mathbf{1_3}$)

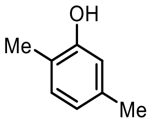

Following **GP4b**, but using BCF (0.5 equiv.) as the Lewis Acid and running the reaction for 30 minutes, $\mathbf{1_1}$ (13 mg, 0.1 mmol, 1.0 equiv.) gave $\mathbf{1_3}$ (69 %) as a solid. ^{1}H NMR ($CDCl_3$, 400 MHz) δ 7.00 (1H, d, J = 7.6 Hz), 6.67 (1H, d, J = 7.6 Hz), 6.61 (1H, s), 4.56 (1H, s), 2.28 (3H,

s), 2.21 (3H, s); ^{13}C NMR ($CDCl_3$, 101 MHz) δ 153.7, 137.2, 130.9, 121.6, 120.5, 115.8, 21.1, 15.4. Data in accordance with the literature.[8]

2,6-Dimethylphenol (1$_4$)

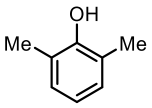

Following **GP4b**, but running the reaction for 2 h, **1$_1$** (13 mg, 0.1 mmol, 1.0 equiv.) gave **1$_4$** (99 %) as a solid. ^{1}H NMR ($CDCl_3$, 400 MHz) δ 7.01 (2H, d, J = 7.5 Hz), 6.79 (1H, t, J = 7.5 Hz), 4.66 (1H, s), 2.28 (6H, s); ^{13}C NMR ($CDCl_3$, 101 MHz) δ 152.3, 128.7, 123.1, 120.3, 15.9. Data in accordance with the literature.[8]

Following **GP4b**, but running the reaction for 2 h, **1$_3$** (13 mg, 0.1 mmol, 1.0 equiv.) gave **1$_4$** (99 %) as a solid

2,4-Dimethylphenol (1$_5$)

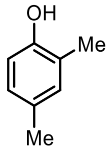

Following **GP4b**, but running the reaction in CH_2Cl_2 (0.05 M), **1$_2$** (13 mg, 0.1 mmol, 1.0 equiv.) gave **1$_5$** (80 %) as a solid. ^{1}H NMR ($CDCl_3$, 400 MHz) δ 6.98 (1H, s), 6.92 (1H, d, J = 8.0 Hz), 6.71 (1H, d, J = 8.0 Hz), 4.78 (1H, s), 2.30 (3H, s), 2.27 (3H, s); ^{13}C NMR ($CDCl_3$, 101 MHz) δ 151.6, 131.8, 130.0, 127.5, 123.6, 114.9, 20.5, 15.8. Data in accordance with the literature.[8]

Following **GP4a**, but running the reaction in CH_2Cl_2 (0.5 M), **1$_3$** (13 mg, 0.1 mmol, 1.0 equiv.) gave **1$_5$** (46 %) as a solid

Following **GP4a**, **1$_6$** (13 mg, 0.1 mmol, 1.0 equiv.) gave **1$_5$** (30 %) as a solid

***o*-Cresol (2$_1$)**

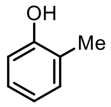

Following **GP4b**, **2$_2$** (11 mg, 0.1 mmol, 1.0 equiv.) gave **2$_1$** (64 %, 15 % rsm) as an oil. ^{1}H NMR ($CDCl_3$, 400 MHz) δ 7.25 – 7.06 (2H, m), 6.91 (1H, td, J = 7.3, 1.2 Hz), 6.81 (1H, d, J = 7.9 Hz), 5.04 (1H, s), 2.30 (3H, s); ^{13}C NMR ($CDCl_3$, 101 MHz) δ 153.8, 131.2, 127.2, 124.0, 120.9, 115.1, 15.8. Data in accordance with the literature.[9]

***p*-Cresol (2_3)**

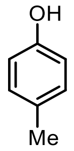

Following **GP4a**, $\mathbf{2_2}$ (11 mg, 0.1 mmol, 1.0 equiv.) gave $\mathbf{2_3}$ (68 %, 8 % rsm) as a solid. ^{1}H NMR ($CDCl_3$, 400 MHz) δ 7.04 (2H, d, J = 8.2 Hz), 6.74 (2H, d, J = 8.2 Hz), 4.82 (1H, s), 2.28 (3H, s); ^{13}C NMR ($CDCl_3$, 101 MHz) δ 153.4, 130.2, 115.2, 20.6. Data in accordance with the literature.[9].

[1,1'-Biphenyl]-4-ol (3_2)

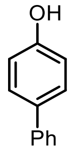

Following **GP4b**, but running the reaction in CH_2Cl_2 (0.05 M), $\mathbf{3_1}$ (15 mg, 0.1 mmol, 1.0 equiv.) gave $\mathbf{3_2}$ (45 %) as a solid. ^{1}H NMR ($CDCl_3$, 400 MHz) δ 7.55 (2H, d, J = 7.0 Hz), 7.49 (2H, d, J = 8.6 Hz), 7.42 (2H, t, J = 7.7 Hz), 7.31 (1H, t, J = 7.3 Hz), 6.92 (2H, d, J = 8.6 Hz), 4.96 (1H, s); ^{13}C NMR ($CDCl_3$, 101 MHz) δ 155.2, 140.9, 134.2, 128.9, 128.5, 126.9, 126.8, 115.8. Data in accordance with the literature.[10]

4'-Methyl-[1,1'-biphenyl]-4-ol (4_2)

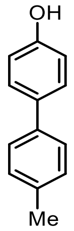

Following **GP4b**, but running the reaction in CH_2Cl_2 (0.025 M) for 48 h, $\mathbf{4_1}$ (18 mg, 0.1 mmol, 1.0 equiv.) gave $\mathbf{4_2}$ (42 %) as a solid. ^{1}H NMR ($CDCl_3$, 600 MHz) δ 7.46 (2H, d, J = 8.0 Hz), 7.43 (2H, d, J = 8.2 Hz), 7.22 (2H, d, J = 8.2 Hz), 6.89 (2H, d, J = 8.6 Hz), 5.17 (1H, s), 2.38 (3H, s); ^{13}C NMR ($CDCl_3$, 151 MHz) δ 155.0, 137.9, 136.5, 133.9, 129.0, 128.2, 126.6, 115.7, 21.1. Data in accordance with the literature.[11]

4'-Fluoro-[1,1'-biphenyl]-4-ol (5_2)

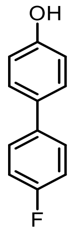

Following **GP4b**, but running the reaction in CH_2Cl_2 (0.025 M) for 48 h, **5**$_1$ (19 mg, 0.1 mmol, 1.0 equiv.) gave **5**$_2$ (55 %) as a solid. ^1H NMR ($CDCl_3$, 600 MHz) δ 7.48 (2H, dd, J = 8.6, 5.3 Hz), 7.42 (2H, d, J = 8.6 Hz), 7.11 (2H, t, J = 4.4 Hz), 6.91 (2H, d, J = 8.6 Hz) 5.12 (1H, s); ^{13}C NMR ($CDCl_3$, 151 MHz) δ 163.4, 161.4, 156.2, 137.0 (d, J = 3.1 Hz), 133.0, 128.7 (d, J = 7.9 Hz), 115.8 (d, J = 11.3 Hz), 115.6; ^{19}F NMR ($CDCl_3$, 564 MHz) δ -116.71. Data in accordance with the literature.[12]

2-Ethyl-6-methylphenol (6_2)

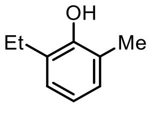

Following **GP4b**, **6**$_1$ (15 mg, 0.1 mmol, 1.0 equiv.) gave **6**$_2$ (29 %) as an oil. ^1H NMR (400 MHz, $CDCl_3$) δ 7.00 – 6.96 (2H, m), 6.79 (1H, t, J = 7.5 Hz), 4.13 (1H, s), 2.63 (2H, q, J = 7.3 Hz), 2.25 (3H, s), 1.24 (3H, t, J = 7.6 Hz); ^{13}C NMR (101 MHz, $CDCl_3$) δ151.8, 132.1, 129.5, 126.3, 123.1, 120.2, 23.9, 15.9, 13.3. Data in accordance with the literature.[13]

Following **GP4b**, **10**$_1$ (15 mg, 0.1 mmol, 1.0 equiv.) gave **6**$_2$ (40 %) as an oil.

4-Methyl-[1,1'-biphenyl]-3-ol (7_2)

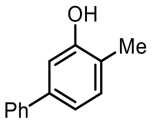

Following **GP4b**, but running the reaction for 24 h, **7**$_1$ (18 mg, 0.1 mmol, 1.0 equiv.) gave **7**$_2$ (50 %, 20 % rsm) as a solid. ^1H NMR ($CDCl_3$, 400 MHz) δ 7.57 (2H, d, J = 7.0 Hz), 7.43 (2H, t, J = 7.6 Hz), 7.34 (1H, t, J = 7.4 Hz), 7.20 (1H, d, J = 7.9 Hz), 7.11 (1H, dd, J = 7.7, 1.8 Hz), 7.03 (1H, d, J = 1.8 Hz), 4.83 (1H, s), 2.31 (3H, s); ^{13}C NMR ($CDCl_3$, 101 MHz) δ 154.1, 140.9, 140.7, 131.5, 128.9, 127.3, 127.1, 122.9, 119.7, 113.8, 15.6. Data in accordance with the literature.[14]

5-Fluoro-2-methylphenol (8_2)

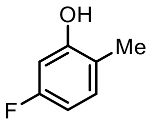

Following **GP4b**, but running the reaction in CH_2Cl_2 (0.05 M), **8_1** (13 mg, 0.1 mmol, 1.0 equiv.) gave **8_2** (49 %, 15 % rsm) as an oil. ^1H NMR ($CDCl_3$, 600 MHz) δ 7.04 (1H, t, J = 7.4 Hz), 6.59 – 6.53 (2H, m), 5.06 (1H, s), 2.20 (3H, s); ^{13}C NMR ($CDCl_3$, 101 MHz) δ 161.9 (d, J = 243.1 Hz), 154.7 (d, J = 11.0 Hz), 131.5 (d, J = 9.6 Hz), 119.4 (d, J = 3.2 Hz), 107.3 (d, J = 21.0 Hz), 102.8 (d, J = 24.4 Hz), 15.2; ^{19}F NMR ($CDCl_3$, 564 MHz) δ -112.92. Data in accordance with the literature.[15]

5-methoxy-2-methylphenol (9_2)

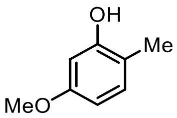

Following **GP4b**, but running the reaction in CH_2Cl_2 (0.05 M) and irradiating at 370 nm, **9_1** (14 mg, 0.1 mmol, 1.0 equiv.) gave **9_2** (60 %) as a solid. ^1H NMR ($CDCl_3$, 400 MHz) δ 7.01 (1H, d, J = 9.0 Hz), 6.42 (2H, dd, J = 8.2, 2.6 Hz), 6.39 (2H, d, J = 2.5 Hz), 4.82 (2H, s), 3.76 (6H, s), 2.18 (8H, d, J = 1.7 Hz); ^{13}C NMR ($CDCl_3$, 101 MHz) δ 159.2, 154.6, 131.3, 115.8, 106.1, 101.6, 55.5, 15.0. Data in accordance with the literature.[16]

2-Ethyl-4-methylphenol (10_2)

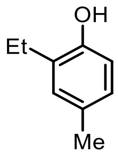

Following **GP4a**, **10_1** (15 mg, 0.1 mmol, 1.0 equiv.) gave **10_2** (18%, 23 % rsm) as an oil. ^1H NMR ($CDCl_3$, 600 MHz) δ 7.02 – 6.96 (1H, m), 6.91 – 6.84 (1H, m),6.57 (1H, d, J = 8.2 Hz), 5.20 (1H, s), 2.59 (2H, q, J = 7.2 Hz), 2.19 (3H, s), 1.16 (3H, t, J = 7.3 Hz); ^{13}C NMR ($CDCl_3$, 151 MHz) δ 151.8, 136.6, 130.6, 126.3, 123.6, 114.9, 28.1, 16.1, 15.9. Data in accordance with the literature.[17]

3-methyl-[1,1'-biphenyl]-2-ol (11_2)

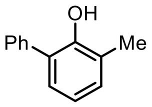

Following **GP4b**, **11_1** (18 mg, 0.1 mmol, 1.0 equiv.) gave **11_2** (57%, 7 % rsm) as an oil. ^1H NMR ($CDCl_3$, 600 MHz) δ 7.55-7.41 (5H, m), 7.11 (1H, d, J = 7.4 Hz), 7.09 (1H, dd, J = 7.6 Hz, J = 1.2 Hz), 6.92 (1H, t, J = 7.5 Hz), 5.23 (s, 1 H), 2.33 (s, 3 H); ^{13}C NMR ($CDCl_3$, 151 MHz) δ 150.7, 137.5, 130.7, 129.5, 129.3, 128.0, 127.9, 127.8, 124.8, S14 120.4, 16.4. Data in accordance with the literature.[18]

2-Fluoro-6-methylphenol (12_2)

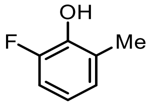

Following **GP4b**, **12_1** (13 mg, 0.1 mmol, 1.0 equiv.) gave **12_2** (34 %, 29 % rsm) as an oil. ^1H NMR ($CDCl_3$, 600 MHz) δ 6.92 – 6.86 (2H, m), 6.73 (1H, td, J = 7.9, 5.4 Hz), 5.62 (1H, s), 2.27 (3H, s); ^{13}C NMR ($CDCl_3$, 151 MHz) δ 151.1 (d, J = 235.7 Hz), 141.9 (d, J = 14.2 Hz), 126.9, 126.1 (d, J = 3.0 Hz), 120.9 (d, J = 6.1 Hz), 112.7 (d, J = 18.4 Hz), 15.4 (d, J = 3.1 Hz); ^{19}F NMR ($CDCl_3$, 564 MHz) δ -141.96. Data in accordance with the literature.[19]

4-Isopropyl-2-methylphenol (13_2)

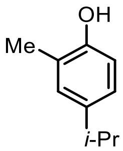

Following **GP4a**, but running the reaction in CH_2Cl_2 (0.0255 M), **13_1** (13 mg, 0.1 mmol, 1.0 equiv.) gave **13_2** (57 %) as a solid. ^1H NMR (400 MHz, $CDCl_3$) δ 6.98 (1H, d, J = 2.3 Hz), 6.94 (1H, dd, J = 8.3, 2.3 Hz), 6.70 (1H, d, J = 8.1 Hz), 4.55 (1H, s), 2.82 (1H, hept, J = 6.8 Hz), 2.24 (3H, s), 1.21 (6H, d, J = 7.0 Hz). ^{13}C NMR ($CDCl_3$, 101 MHz) δ 151.9, 141.4, 129.2, 124.9, 123.5, 114.9, 33.4, 24.4, 16.0. Data in accordance with the literature.[20]

2-Isopropyl-6-methylphenol (13$_3$)

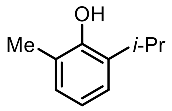

Following **GP4b**, but running the reaction for 2 h, **14$_1$** (13 mg, 0.1 mmol, 1.0 equiv.) gave **13$_3$** (86 %) as an oil. ^{1}H NMR (400 MHz, $CDCl_3$) δ 7.10 – 7.02 (1H, m), 6.98 (1H, d, *J* = 7.5 Hz), 6.84 (1H, t, *J* = 7.6 Hz), 4.66 (1H, s), 3.20 (1H, hept, *J* = 6.8 Hz), 2.26 (3H, s), 1.27 (6H, d, *J* = 6.9 Hz); ^{13}C NMR (101 MHz, $CDCl_3$) δ 151.2, 133.9, 128.3, 124.1, 123.0, 120.6, 27.2, 22.8, 16.1. HRMS (EI): found M^+ 150.1039, $C_{10}H_{14}O$ requires 150.1039.

Following **GP4b**, **13$_1$** (1,5 g, 10 mmol, 1.0 equiv.) gave **13$_3$** (78 %) as an oil.

2-Isopropyl-4-methylphenol (14$_2$)

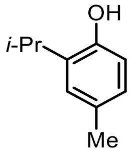

Following **GP4a**, but running the reaction in CH_2Cl_2 (0.025 M), **14$_1$** (13 mg, 0.1 mmol, 1.0 equiv.) gave **14$_2$** (52 %) as a solid. ^{1}H NMR ($CDCl_3$, 400 MHz) δ 7.02 (1H, d, *J* = 2.2 Hz), 6.91 – 6.84 (1H, m), 6.66 (1H, d, *J* = 8.0 Hz), 4.64 (1H, s), 3.20 (1H, hept, *J* = 6.9 Hz), 2.30 (3H, s), 1.27 (6H, d, *J* = 6.9 Hz); ^{13}C NMR ($CDCl_3$, 101 MHz) δ 150.6, 134.3, 130.2, 127.1 (2C), 115.3, 27.1, 22.8, 20.9. Data in accordance with the literature.[21]

4-Methyl-6-pentylphenol (15$_2$)

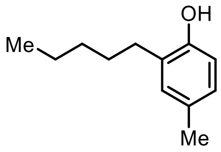

Following **GP4a**, **15$_1$** (18 mg, 0.1 mmol, 1.0 equiv.) gave **15$_2$** (42 %) as a solid. R_f 0.34 [pentane:EtOAc (95:5)]. ^{1}H NMR ($CDCl_3$, 600 MHz) δ 6.93 (1H, d, *J* = 2.1 Hz), 6.87 (1H, dd, *J* = 8.0, 2.3 Hz), 6.66 (1H, d, *J* = 8.0 Hz), 4.49 (1H, s), 2.74 – 2.48 (2H, m), 2.26 (3H, s), 1.70 – 1.53 (2H, m), 1.38 – 1.26 (4H, m), 0.97 – 0.70 (3H, m); ^{13}C NMR ($CDCl_3$, 151 MHz) δ 151.2, 130.9, 130.0, 128.5, 127.5, 115.2, 31.9, 30.1, 29.8, 22.7, 20.7, 14.2. HRMS (EI): found M^+ 178.1352, $C_{12}H_{18}O$ requires 178.1352.

2-Methyl-6-pentylphenol (15$_3$)

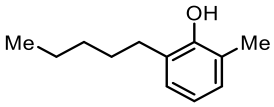

Following **GP4b**, **15$_1$** (18 mg, 0.1 mmol, 1.0 equiv.) gave **15$_3$** (86 %) as a solid. ^{1}H NMR (400 MHz, CDCl$_3$) δ 7.00 (2H, d, J = 7.5 Hz), 6.80 (1H, t, J = 7.5 Hz), 4.63 (1H, s), 2.61 (2H, d, J = 7.9 Hz), 2.28 (3H, s), 1.70 – 1.60 (2H, m), 1.38 (4H, h, J = 3.8 Hz), 0.99 – 0.89 (3H, m); ^{13}C NMR (101 MHz, CDCl$_3$) δ 151.9, 128.6, 128.0, 127.9, 123.2, 120.4, 31.9, 30.2, 29.6, 22.7, 16.1, 14.2. Data in accordance with the literature.[22]

4-Ethyl-2-methylphenol (16$_2$)

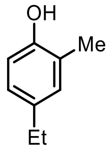

Following **GP4b**, **16$_1$** (15 mg, 0.1 mmol, 1.0 equiv.) gave **16$_2$** (70 %) as an oil. ^{1}H NMR (CDCl$_3$, 600 MHz) δ 6.94 (1H, s), 6.90 (1H, d, J = 8.2 Hz), 6.69 (1H, d, J = 8.2 Hz), 4.63 (1H, s), 2.54 (2H, q, J = 7.6 Hz), 2.23 (3H, s), 1.19 (3H, t, J = 7.6 Hz); ^{13}C NMR (CDCl$_3$, 151 MHz) δ 151.8, 136.6, 130.6, 126.3, 123.6, 114.9, 28.1, 16.1, 15.9. Data in accordance with the literature.[23]

3-Methyl-[1,1'-biphenyl]-4-ol (17$_2$)

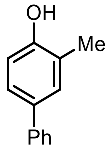

Following **GP4b**, but running the reaction for 24 h, **17$_1$** (18 mg, 0.1 mmol, 1.0 equiv.) gave **17$_2$** (35 %, 36 % rsm) as a solid. ^{1}H NMR (CDCl$_3$, 600 MHz) δ 7.57 (2H, d, J = 8.3 Hz), 7.46 – 7.39 (3H, m), 7.38 – 7.29 (2H, m), 6.86 (1H, d, J = 8.2 Hz), 5.50 (1H, s), 2.35 (3H, s); ^{13}C NMR (CDCl$_3$, 151 MHz) δ 153.7, 141.1, 133.9, 129.9, 128.8, 126.8, 126.7, 125.8, 124.4, 115.3, 16.1. Data in accordance with the literature.[24]

5-Methyl-[1,1'-biphenyl]-2-ol (18_2)

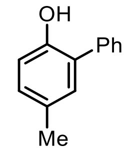

Following **GP4b**, but running the reaction in CH_2Cl_2 (0.05 M), **18_1** (18 mg, 0.1 mmol, 1.0 equiv.) gave **18_2** (70 %) as a solid. ^{1}H NMR ($CDCl_3$, 600 MHz) δ 7.54 – 7.46 (5H, m), 7.09 – 7.07 (2H, m), 6.90 (1H, d, J = 8.7 Hz), 5.13 (1H, s), 2.34 (3H, s); ^{13}C NMR ($CDCl_3$, 101 MHz) δ 150.3, 137.4, 130.8, 130.1, 129.7, 129.3, 129.2, 128.0, 127.8, 115.8, 20.6. Data in accordance with the literature.[25]

2-Chloro-4,6-dimethylphenol (19_2)

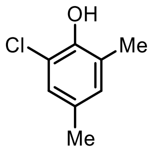

Following **GP4b**, **19_1** (16 mg, 0.1 mmol, 1.0 equiv.) gave **19_2** (90 %) as a solid. ^{1}H NMR ($CDCl_3$, 600 MHz) δ 7.05 (1H, s), 6.80 (1H, s), 5.29 (1H, s), 2.19 (3H, s), 2.16 (3H, s); ^{13}C NMR ($CDCl_3$, 151 MHz) δ 147.4, 130.5, 129.5, 126.5, 125.6, 119.2, 20.4, 16.3. Data in accordance with the literature.[26]

2,5-Dimethyl-[1,1'-biphenyl]-4-ol (20_2)

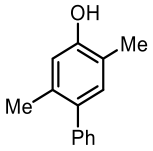

Following **GP4b**, but irradiating at 350 nm, **20_1** (20 mg, 0.1 mmol, 1.0 equiv.) gave **20_2** (47 %) as a solid. ^{1}H NMR ($CDCl_3$, 600 MHz) δ 7.41 – 7.36 (2H, m), 7.32 – 7.27 (3H, m), 6.99 (1H, s), 6.69 (1H, s), 5.01 (1H, s), 2.24 (3H, s), 2.20 (3H, s); ^{13}C NMR ($CDCl_3$, 151 MHz) δ 153.1, 141.8, 134.6, 134.2, 132.5, 129.5, 128.1, 126.5, 121.0, 116.7, 20.2, 15.4. Data in accordance with the literature.[27]

3,5-Dimethyl-[1,1'-biphenyl]-4-ol (**20$_3$**)

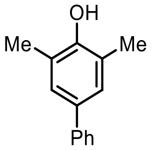

Following **GP4b**, **20$_1$** (20 mg, 0.1 mmol, 1.0 equiv.) gave **20$_3$** (14 %, 20 % **20$_2$**) as a solid. ^{1}H NMR ($CDCl_3$, 600 MHz) δ 7.58 (2H, d, J = 7.2 Hz), 7.46 (2H, t, J = 7.6 Hz), 7.29 (1H, t, J = 7.5 Hz), 7.27 (2H, s), 4.62 (1H, s), 2.29 (6H, s); ^{13}C NMR ($CDCl_3$, 151 MHz) δ 151.0, 141.5, 132.9, 128.4, 127.1, 126.5, 126.3, 122.9, 15.8. Data in accordance with the literature.[28]

4-Chloro-2,5-dimethylphenol (**21$_2$**)

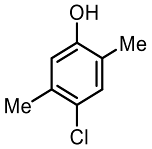

Following **GP4b**, but running the reaction at 370 nm, **21$_1$** (16 mg, 0.1 mmol, 1.0 equiv.) gave **21$_2$** (80 %, 11% **21$_3$**, 11 % rsm) as a solid. ^{1}H NMR ($CDCl_3$, 600 MHz) δ 7.08 (1H, s), 6.64 (1H, s), 4.56 (1H, s), 2.28 (3H, s), 2.18 (3H, s); ^{13}C NMR ($CDCl_3$, 151 MHz) δ 152.3, 134.5, 131.0, 128.2, 122.9, 117.4, 19.8, 15.3. HRMS (EI): found M^+ 156.0335, C_8H_9OCl requires 156.0336.

4-Chloro-2,6-dimethylphenol (**21$_3$**)

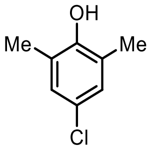

Following **GP4b**, but running the reaction at 370 nm, **21$_1$** (16 mg, 0.1 mmol, 1.0 equiv.) gave **21$_3$** (11 %, 80% **21$_2$**, 11 % rsm) as a solid. ^{1}H NMR ($CDCl_3$, 400 MHz) δ 6.96 (2H, s), 4.54 (1H, s), 2.22 (3H, s), 2.20 (3H, s); ^{13}C NMR ($CDCl_3$, 101 MHz) δ 150.9, 128.6, 127.9, 124.7, 15.9. Data in accordance with the literature.[29]

2,4,5-Trimethylphenol (**22$_2$**)

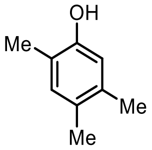

Following **GP4a**, **22$_1$** (15 mg, 0.1 mmol, 1.0 equiv.) gave **22$_2$** (26 %) as a solid. ^{1}H NMR ($CDCl_3$, 600 MHz) δ 6.88 (1H, s), 6.59 (1H, s), 5.16 (1H, s), 2.27 (3H, s), 2.26 (3H, s), 2.17

(3H, s); ^{13}C NMR ($CDCl_3$, 151 MHz) δ 151.7, 135.2, 132.2, 128.7, 120.6, 116.4, 19.5, 18.8, 15.3. Data in accordance with the literature.[30]

2,3,6-Trimethylphenol (22_3)

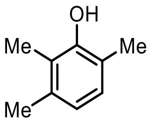

Following **GP4a**, but running the reaction for 2 h, **22_1** (15 mg, 0.1 mmol, 1.0 equiv.) gave **22_3** (99 %) as a solid. ^{1}H NMR ($CDCl_3$, 600 MHz) δ 6.86 (1H, d, J = 7.6 Hz), 6.67 (1H, d, J = 7.6 Hz), 4.64 (1H, s), 2.25 (3H, s), 2.22 (3H, s), 2.16 (3H, s); ^{13}C NMR ($CDCl_3$, 151 MHz) δ 152.0, 135.7, 127.5, 121.9, 121.8, 120.3, 20.1, 15.9, 11.7. Data in accordance with the literature.[31]

2,4,6-Trimethylphenol (22_4)

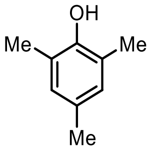

Following **GP4a**, **22_3** (15 mg, 0.1 mmol, 1.0 equiv.) gave **22_4** (92 %) as a solid. ^{1}H NMR ($CDCl_3$, 400 MHz) δ 6.80 (2H, s), 4.48 (1H, s), 2.23 (3H, s), 2.23 (6H, s); ^{13}C NMR ($CDCl_3$, 101 MHz) δ 150.0, 129.4, 129.2, 122.9, 20.5, 15.9. Data in accordance with the literature.[32]

1-Methoxy-2,4-dimethylbenzene (23_2)

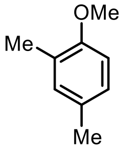

Following **GP4a**, but using TfOH (2.0 equiv.) as the additive, **23_1** (14 mg, 0.1 mmol, 1.0 equiv.) gave **23_2** (42 %, 24 % rsm) as a solid. ^{1}H NMR ($CDCl_3$, 600 MHz) δ 6.96 – 6.91 (2H, m), 6.71 (1H, d, J = 9.0 Hz), 3.81 (3H, s), 2.25 (3H, s), 2.18 (3H, s); ^{13}C NMR ($CDCl_3$, 151 MHz) δ 155.8, 131.6, 129.0, 127.0, 126.3, 110.0, 55.5, 20.5, 16.2. Data in accordance with the literature.[33]

2-Methoxy-1,3-dimethylbenzene (23₃)

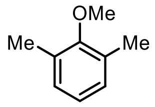

Following **GP4b**, but using TfOH (5.0 equiv.) as the additive, **23₁** (14 mg, 0.1 mmol, 1.0 equiv.) gave **23₃** (40 %, 31 % rsm) as a solid. ^{1}H NMR ($CDCl_3$, 600 MHz) δ 7.00 (2H, d, J = 7.2 Hz), 6.93 – 6.88 (1H, m), 3.72 (3H, s), 2.28 (6H, s); ^{13}C NMR ($CDCl_3$, 151 MHz) δ 157.6, 130.4, 128.9, 123.4, 59.7, 15.9. Data in accordance with the literature.[34]

7.1 Additional Substrates

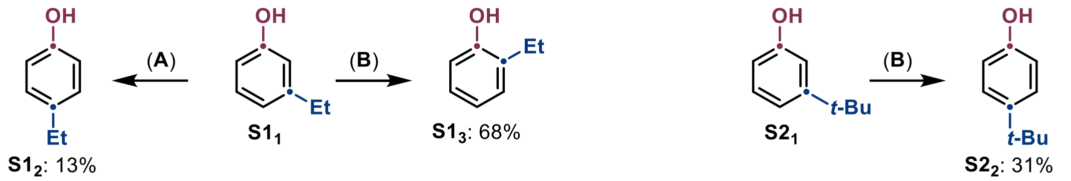

2-ethylphenol (S1$_2$)

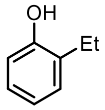

Following **GP4b**, but running the reaction in CH_2Cl_2 (0.05 M), **S1$_1$** (12 mg, 0.1 mmol, 1.0 equiv.) gave **S1$_2$** (68 %, 18 % **S1$_3$**) as a solid. ^{1}H NMR ($CDCl_3$, 600 MHz) 7.15 – 7.01 (2H, m), 6.89 – 6.82 (1H, m), 6.73 (1H, d, *J* = 8.0 Hz), 4.61 (1H, s), 2.62 (2H, q, *J* = 7.6 Hz), 1.22 (3H, t, *J* = 7.6 Hz); ^{13}C NMR ($CDCl_3$, 151 MHz) 153.6, 130.0, 129.6, 127.3, 21.2, 115.0, 23.0, 14.3. Data in accordance with the literature.[35]

4-ethylphenol (S1$_3$)

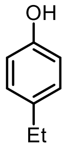

Following **GP4a**, but running the reaction in CH_2Cl_2 (0.05 M), **S1$_1$** (12 mg, 0.1 mmol, 1.0 equiv.) gave **S1$_3$** (13 %, 62 % rsm) as a solid. ^{1}H NMR ($CDCl_3$, 600 MHz) 7.09 (2H, d, *J* = 8.5 Hz), 6.72 (2H, d, *J* = 8.5 Hz), 2.51 (2H, q, *J* = 7.6 Hz), 1.22 (3H, t, *J* = 7.6 Hz); ^{13}C NMR ($CDCl_3$, 151 MHz) 153.5, 136.7, 129.0, 115.1, 27.6, 15.8. Data in accordance with the literature.[35]

4-(tert-butyl)phenol (S2$_2$)

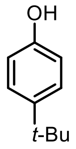

Following **GP4b**, **S2$_1$** (14 mg, 0.1 mmol, 1.0 equiv.) gave **S2$_2$** (31 %, 10 % rsm) as a solid. ^{1}H NMR ($CDCl_3$, 400 MHz) δ 7.25 (1H, d, *J* = 8.7 Hz), 6.77 (1H, d, *J* = 8.8 Hz), 4.86 (1H, s), 1.29

(6H, s); ^{13}C NMR ($CDCl_3$, 101 MHz) δ 153.3, 143.7, 126.6, 114.9, 34.2, 31.7. Data in accordance with the literature.[36]

8 Computational Details

All calculations have been carried out in the framework of density functional theory (DFT) using the CAM-B3LYP exchange-correlation functional.[37] Unless indicated otherwise, the *Gaussian* 16 [43] software was employed. Here we provide a list of the corresponding level of theory used for different calculation

- **Ground-state geometry optimizations and harmonic vibrational frequencies**: For both potential-energy minima and transition states, CAM-B3LYP functional and cc-pVDZ[38] basis set in gas phase. Minima are characterized by all-real vibrational frequencies, whereas transition states show one single imaginary frequency that corresponds to the reaction normal mode.
- **Intrinsic reaction coordinate (IRC) calculations**: CAM-B3LYP functional and cc-pVDZ basis set in gas phase. The default algorithm was employed, and analytic second derivatives were recalculated every 10 steps.
- **Single-point energy calculations**: CAM-B3LYP functional and aug-cc-pVTZ basis set including solvent effects (DCM) with the SMD model.[39]
- **Vertical excitation energies**: Within the time-dependent (TD) DFT formalism[40], TD-CAM-B3LYP functional and aug-cc-pVTZ basis set including solvent effects (DCM) with the SMD model.
- **Optimization of S_1/S_0 conical intersections**: Using *ORCA 5.0.3*[41], TD-CAM-B3LYP functional and cc-pVDZ basis set in gas phase.
- **Non-adiabatic molecular dynamics (NAMD)**: Using *TURBOMOLE 7.7.0*[42], TD-CAM-B3LYP functional and cc-pVDZ basis set in gas phase. The Tully's fewest-switches surface hopping algorithm was employed. For each system, 100 initial geometries were generated from a Wigner sampling using the CAM-B3LYP/cc-pVDZ harmonic vibrational frequencies. For each of those geometries, the initial velocities were randomly generated using the *mdprep* module of *TURBOMOLE 7.7.0*, corresponding to a Maxwell-Boltzmann distribution at a selected temperature of 298.15 K. Using a time step of 40 a.u. (*ca.* 1 fs), all trajectories were propagated for 512 steps (*ca.* 500 fs). From the combined trajectories, the excited-state half-life was estimated as the time in which half of the trajectories reached the ground state, and the photoisomerization quantum yield corresponds to the sum of all trajectories for which

the corresponding C-C bond distance went below 1.70 Å at some point of the trajectory, regardless of subsequent events.

UV-Vis absorption spectra: For each of the geometries generated with the Wigner sampling, the vertical excitation energies for the lowest 10 excited singlet states were calculated at the TD-CAM-B3LYP/aug-cc-pVTZ/SMD(DCM) level of theory. Then, for each geometry an absorption spectrum was generated from the excitation energies and oscillator strengths by convolution to Gaussian functions using a half-width of 0.2 eV. Finally, the (semiclassical) absorption spectrum was obtained as the sum of all the individual absorption spectra.

9 Computational Studies

We carried out several computational simulations to further understand the mechanism of the permutation of phenols. In particular, we were interested in assessing why the permutation of **2_2** is wavelength-dependent and can lead to the formation of either the ortho or the para isomers, whereas the permutation of **3_1** always leads to the para isomer, regardless of the irradiation wavelength. We first started by elucidating the effect of the Lewis acid on the acidity of the OH group (Figure S8). Here we used $AlCl_3$ as model to reduce the computational effort, as we observed a nearly identical reactivity as if $AlBr_3$ is used instead. The self-protonation of phenols has a very large reaction energy ($\Delta G^0 > 55$ kcal mol^{-1}). Coordination of $AlCl_3$ to the OH group through one of the oxygen lone-pairs decreases the basicity of the ring ($\Delta G^0 > 70$ kcal mol^{-1}). In contrast, the lability of the O-H bond is highly increased, leading to the formation of a phenolate-$AlCl_3$ anion and a free protonated phenol with a ΔG^0 *ca.* 50 kcal mol^{-1} lower than the self-protonation. Despite being still endergonic, AlC13-mediated proton transfer is much more favorable than self-protonation.

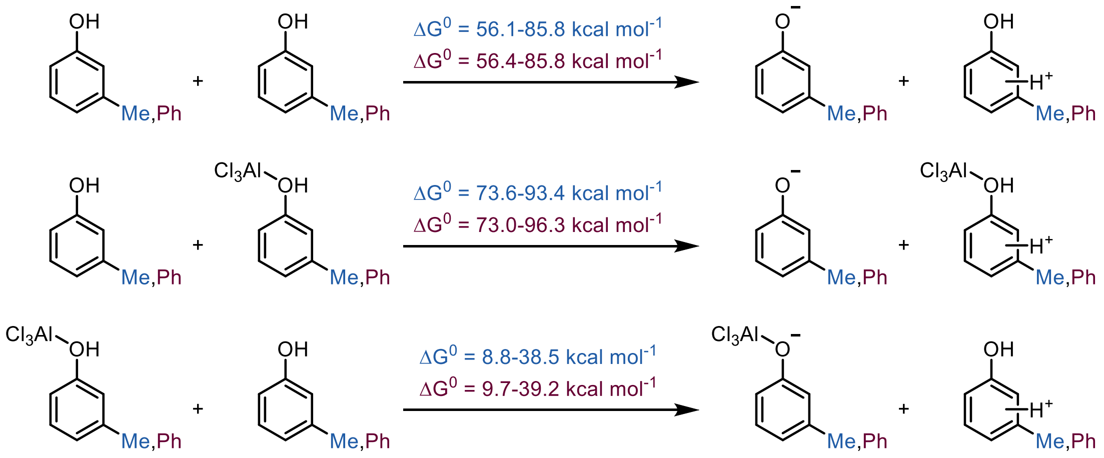

Figure S8. TD-CAM-B3LYP/aug-cc-pVTZ/SMD(DCM) Gibbs free energies for the possible self-protonation reactions of **2_2** (blue) and **3_1** (purple) mediated by $AlCl_3$.

It is worth noting that we provided ranges for the ΔG^0 values as there are multiple potential sites for protonation, including the OH group and each of the six carbon atoms of the phenyl ring (we did not consider protonation of the Ph substituent of **3_1**). Figure S9 shows the relative stabilities of the possible protonated isomers. Protonation at the C_4 position is the most favorable regardless of the substituent, followed by C_2/C_6 at *ca.* 2-3 kcal mol^{-1}, which is consistent with the *ortho*- and *para*-directing effect of the OH group. All the other isomers lie much higher in energy.

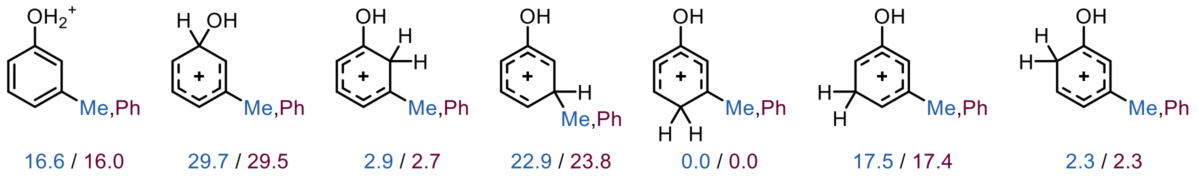

Figure S9. TD-CAM-B3LYP/aug-cc-pVTZ/SMD(DCM) relative Gibbs free energies (in kcal mol^{-1}) for the possible isomers of the protonated species of **2**$_2$ (blue) and **3**$_1$ (red).

Therefore, following coordination to $AlCl_3$, a small protonation of the C_2 and C_4 positions is expected, with the C_2-protonated species being in a much lower proportion due to their higher relative energies. As we observed wavelength-dependent photoisomerization selectivities in the experiments, we decided to calculate the UV-vis absorption spectra of the neutral, C_2-protonated and C_4-protonated species of the two model phenols, which are shown in Figure S10. For **2**$_2$, no absorption is observed above 400 nm. Focusing on the irradiation wavelengths, at 310 nm the absorption is dominated by the C_4-protonated species, whereas the absorption at 390 nm corresponds exclusively to the C_2-protonated species. In contrast, irradiation of **3**$_1$ at both 310 and 390 nm targets the C_4-protonated isomer, as the absorption spectra of both protonated isomers of **3**$_1$ is red-shifted *ca.* 100 nm with respect to **2**$_2$. In all cases, the lowest-energy absorption band corresponds to the excitation to the S_1 state, of π,π* character.

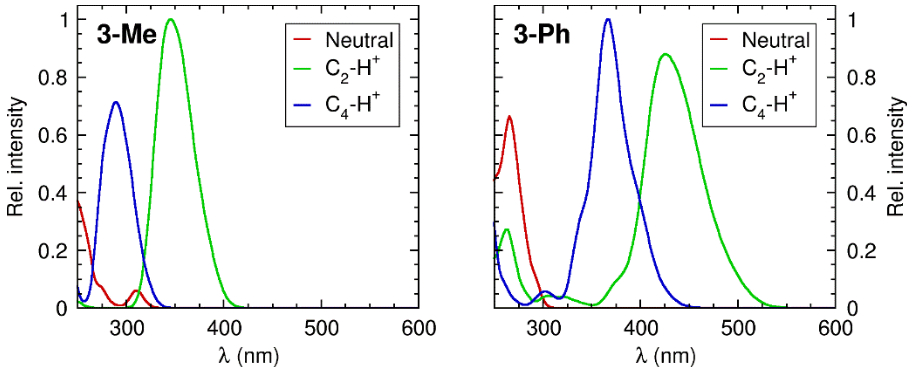

Figure S10. TD-CAM-B3LYP/aug-cc-pVTZ/SMD(DCM) UV-vis absorption spectra of **2**$_2$ (left) and **3**$_1$ (right) phenols, for their neutral (red), C_2-protonated (green) and C_4-protonated (blue) forms.

Next, we wanted to investigate why the $^1\pi,\pi^*$ state evolves towards the formation of different isomers depending on the protonation site. We carried out NAMD simulations (Figure S11) to study the excited-state dynamics of **2**$_2$ following 310 nm irradiation (excitation of the C_4-protonated species) and 390 nm irradiation (excitation of the C_2-protonated species). At the

beginning of the simulations, t = 0 fs, all the trajectories start in the S_1 state. With time, internal conversion leads to the repopulation of the ground state. Regardless of the irradiated protonated species, ground-state repopulation is completed 500 fs after the excitation. However, the rate at which it takes place differs between these two isomers. The excited-state half-life, i.e. the time at which the populations of the S_0 and S_1 states are both equal to 0.5 (50%), is 163 fs for **2**$_2$-C_2H^+ and 78 fs for **2**$_2$-C_4H^+, two times lower. Moreover, the de-excitation of **2**$_2$-C_4H^+ is more sudden, as it mostly takes place in *ca*. 50 fs.

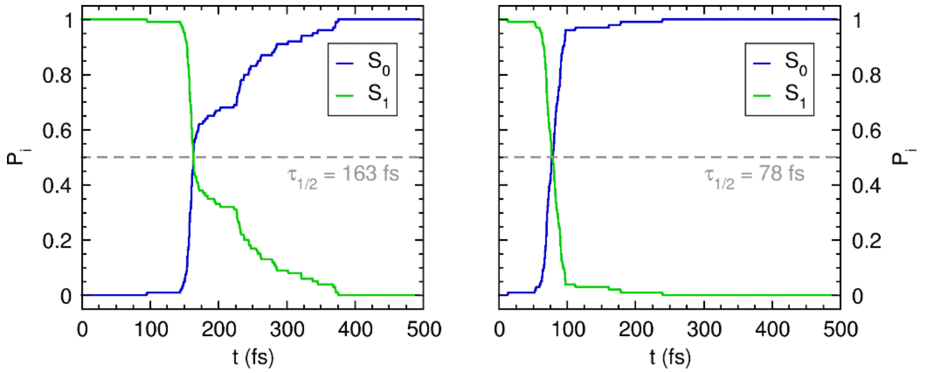

Figure S11. Time evolution of the S_0 (blue) and S_1 (green) populations for the excited-state relaxation of the C_2-protonated (left) and C_4-protonated (right) forms of **2**$_2$, obtained from 100 trajectories of TD-CAM-B3LYP/cc-pVDZ NAMD simulations.

The analysis of the internal conversion points revealed that the geometries of the conical intersections are characterized by the loss of planarity of the phenyl ring and the first stages of the formation of a bond between the carbon atoms adjacent to the CH_2 group (C_1 and C_3 for **2**$_2$-C_2H^+, and C_3 and C_5 for **2**$_2$-C_4H^+). Therefore, we tracked the evolution of these interatomic distances for all the calculated trajectories, which allowed us to obtain estimates for the photoisomerization quantum yields. For **2**$_2$-C_4H^+, 79% of the trajectories led to the formation of the carbon-carbon bond, leading to the bicyclo[3.1.0]hexyl intermediate. In contrast, the photoisomerization quantum yield of **2**$_2$-C_2H^+ is much lower, about 18%, and once the bicyclic intermediate is formed the reversion to the planar species is more facile. Crucially, we did not detect the formation of undesired photoproducts in any of the 200 trajectories, which rules out side reactions. This means that the differences in quantum yields are only relevant for the sake of irradiation efficiency and not for reaction yields.

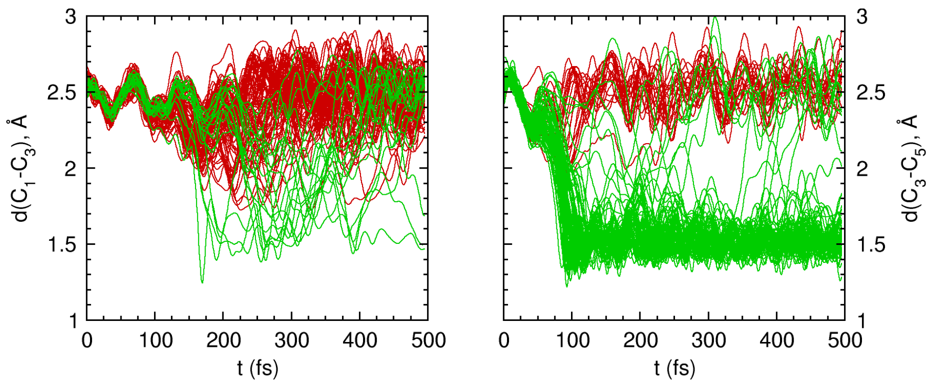

Figure S12. Evolution of the key carbon-carbon distances during the excited-state relaxation of the C_2-protonated (left) and C_4-protonated (right) forms of **2_2**, obtained from TD-CAM-B3LYP/cc-pVDZ NAMD simulations. The green lines represent the trajectories that undergo photoisomerization to the closed form, i.e. the distance goes below 1.70 Å at some point after internal conversion. The red lines represent the trajectories that directly return to the ground-state minimum.

As the forming carbon-carbon bond always takes place so it generates an exocyclic CH_2, the possible phenyl isomer (*ortho* or *para*) that could arise following rearrangement of the bicyclic intermediate is unique for each protonated species. For **2_2**-C_2H^+ (Figure S13 left), only the ortho isomer can be formed from one of the bicycles, as all the other ones would regenerate the initial meta isomer. This bicyclic structure has a low activation barrier for its formation (8.2 kcal mol^{-1}) and is considerably more stable than the isomer formed in the photochemical ring closure (-24.9 kcal mol^{-1}).

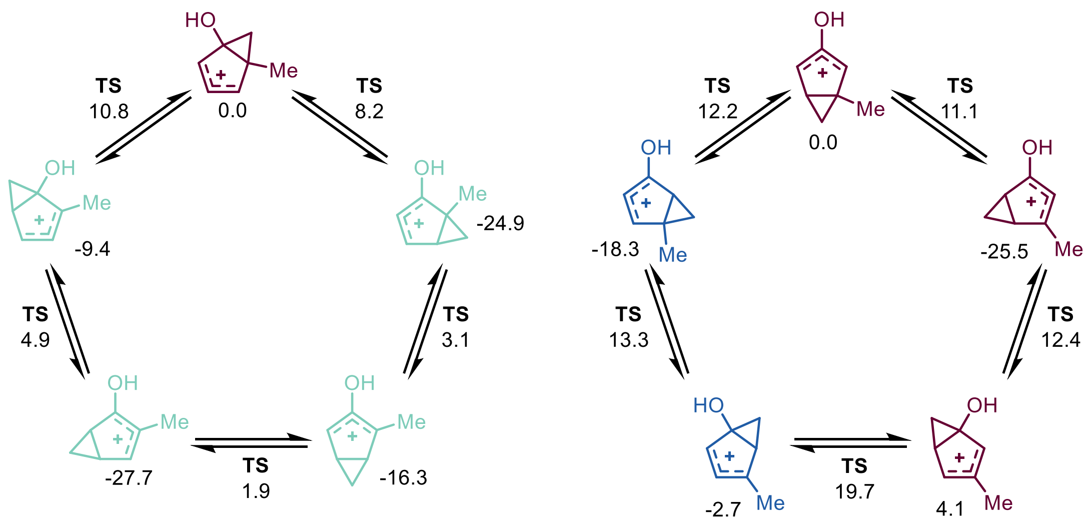

Figure S13. Computed pathways for the thermal isomerization of the closed forms of the C_2-protonated (left) and C_4-protonated (right) forms of **2_2**. TD-CAM-B3LYP/aug-cc-

pVTZ/SMD(DCM) Gibbs free energies (in kcal mol^{-1}) are given relative to the isomer formed during the photochemical ring closure, which is drawn at the top in both schemes. The color of the structure determines which isomer would rise following cleavage of the bridge bond: *ortho* in green, *meta* (original) in red, *para* in blue.

The situation is slightly different for **2**$_2$-C_4H^+ (Figure S13 right). The generation of the *para*-forming bicyclic isomer is not the most favorable rearrangement, as clockwise migration of the CH2 group has a slightly lower barrier (11.1 kcal mol^{-1}) than the counterclockwise migration (12.2 kcal mol^{-1}). Moreover, that isomer is 7.2 kcal mol-1 more stable. However, the only implication these features will have is to reduce the effective photoisomerization quantum yield, as the regeneration of the meta phenol is more favorable during these thermal rearrangement steps.

9.1 Summary

A complete summary of the reaction mechanism can be found in Figure **S14** below. First, **2_2** is protonated by the **2_2**-$AlCl_3$ complex, leading to **A3** or **A1**. These species absorb light of a specific wavelength to populate the S1 state, which lead in both cases to a conical intersection (CI) to the ground state in a barrierless way. Internal conversion triggers the ring closure to **B1** and **B6** respectively. From here, a series of thermal steps could occur, involving rearrangement via 1,2-methylene shift, ring opening, and rearomatization mediated by the $AlCl_3$-phenolate anion. An alternative approach, as discussed in the manuscript, would require 1,2-methylene shift, deprotonation, photoexcitation to the triplet state and ring opening.

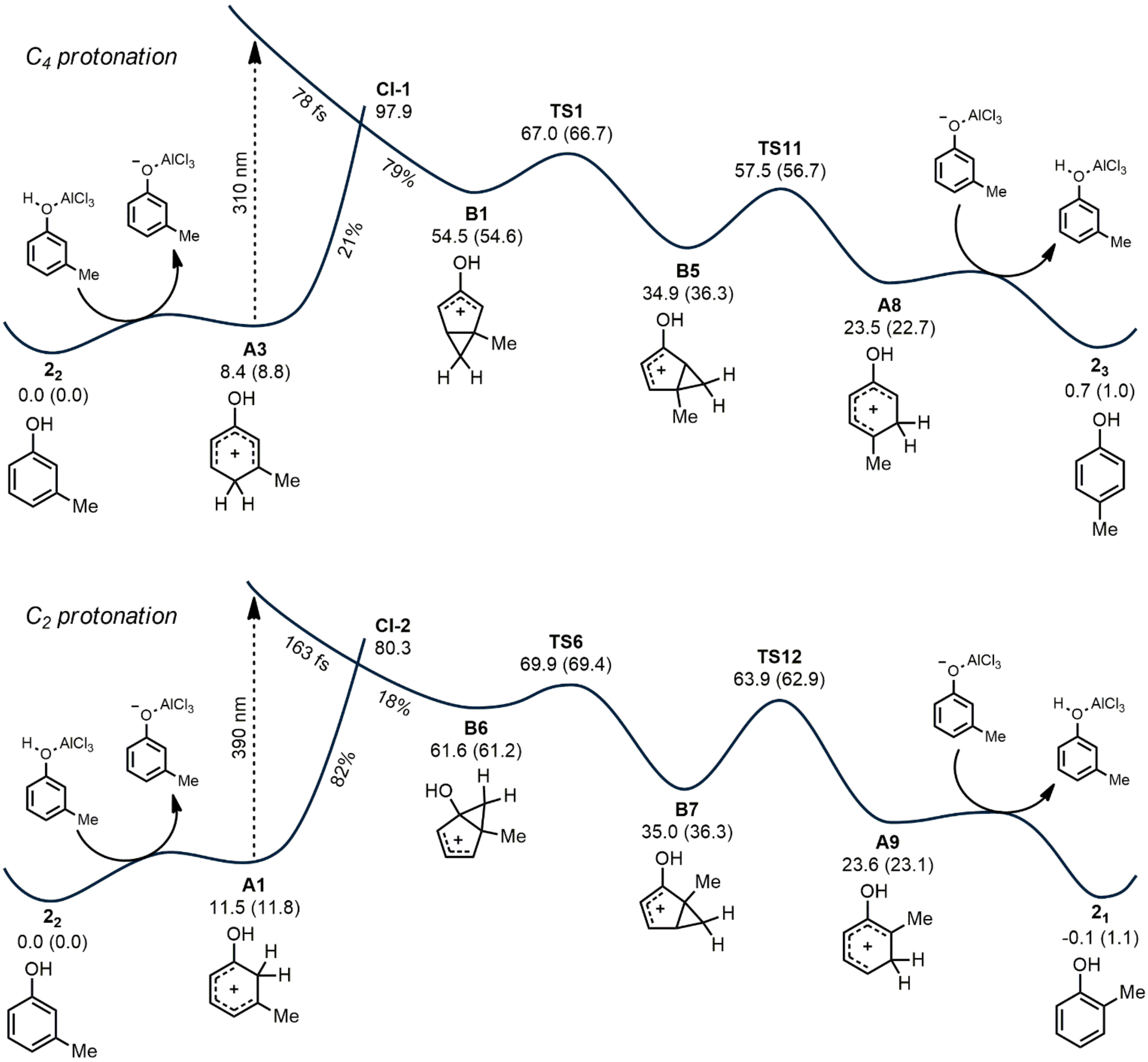

Figure S14. Complete mechanism for the permutation of **2_2** into **2_3** (top) and **2_1** (bottom).

10 Mechanism for Dimethyl Phenols (1_1-1_6)

A summary of the reaction mechanism for the dimethyl phenols **1_1-1_6** is provided in this section.

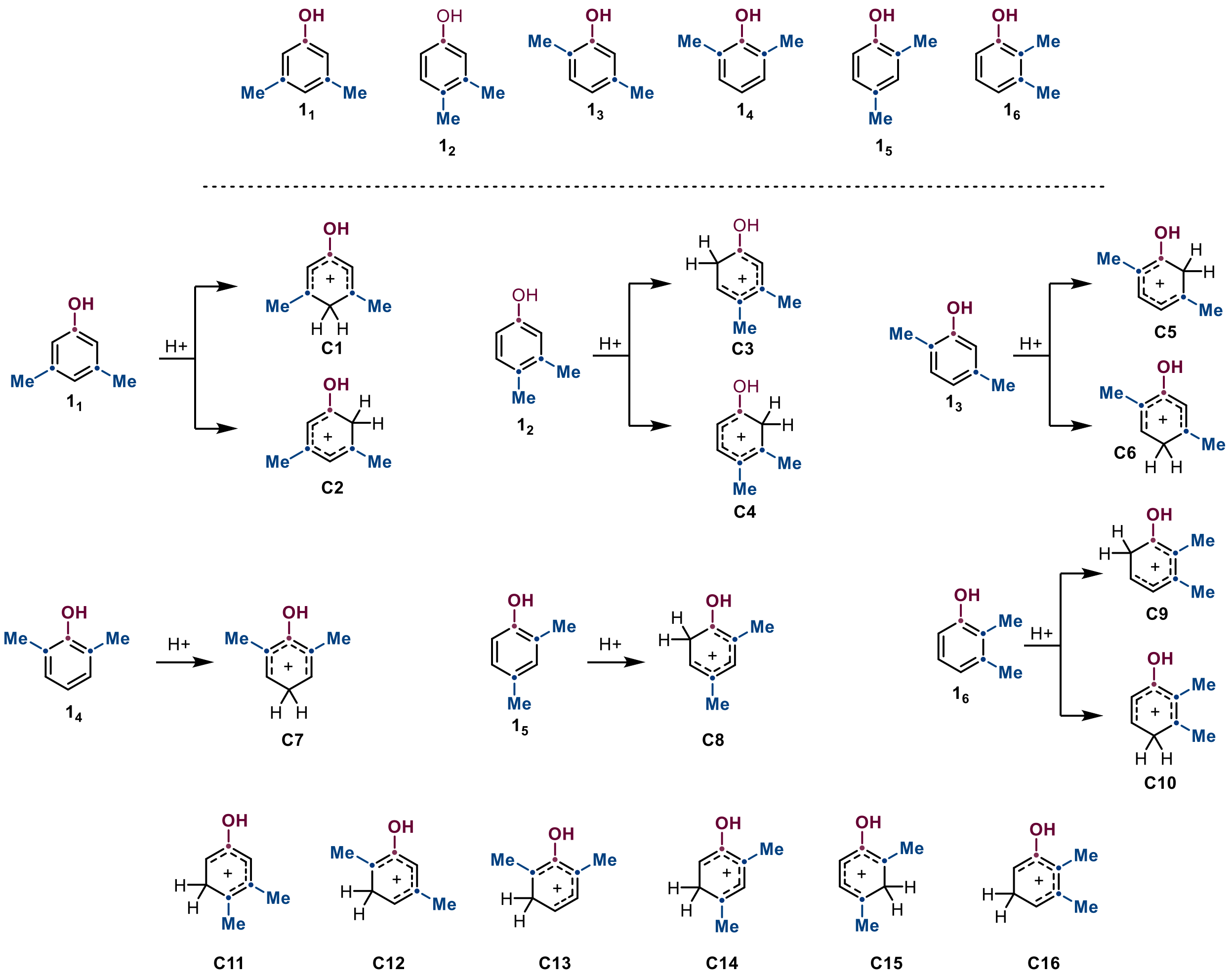

Figure S15. Protonated forms of dimethyl phenols **1_1-1_6** leading to arenium ions **C1-C10** and other additional less stable arenium ions **C11-C16.**

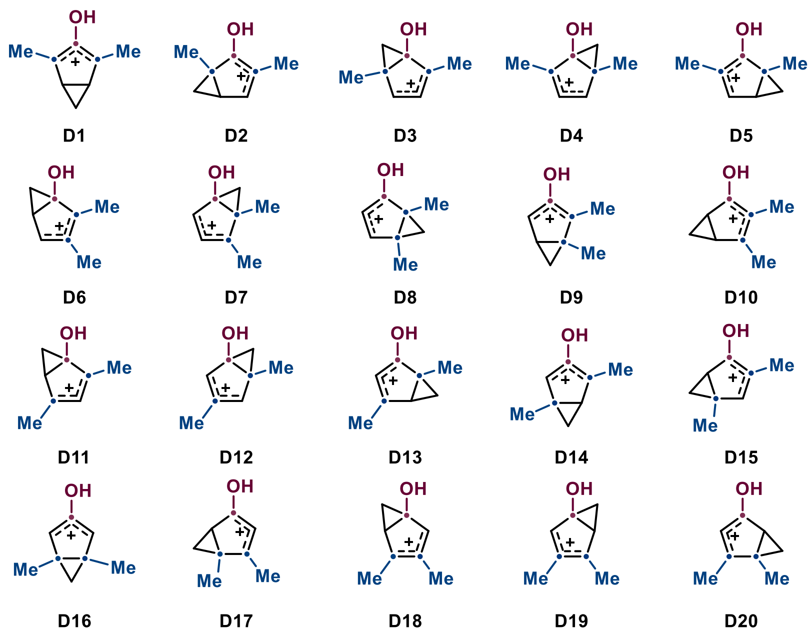

Figure S16. Possible bicyclic cation intermediates **D1-D2** formed from the 4π-electrocyclization of the corresponding arenium ion.

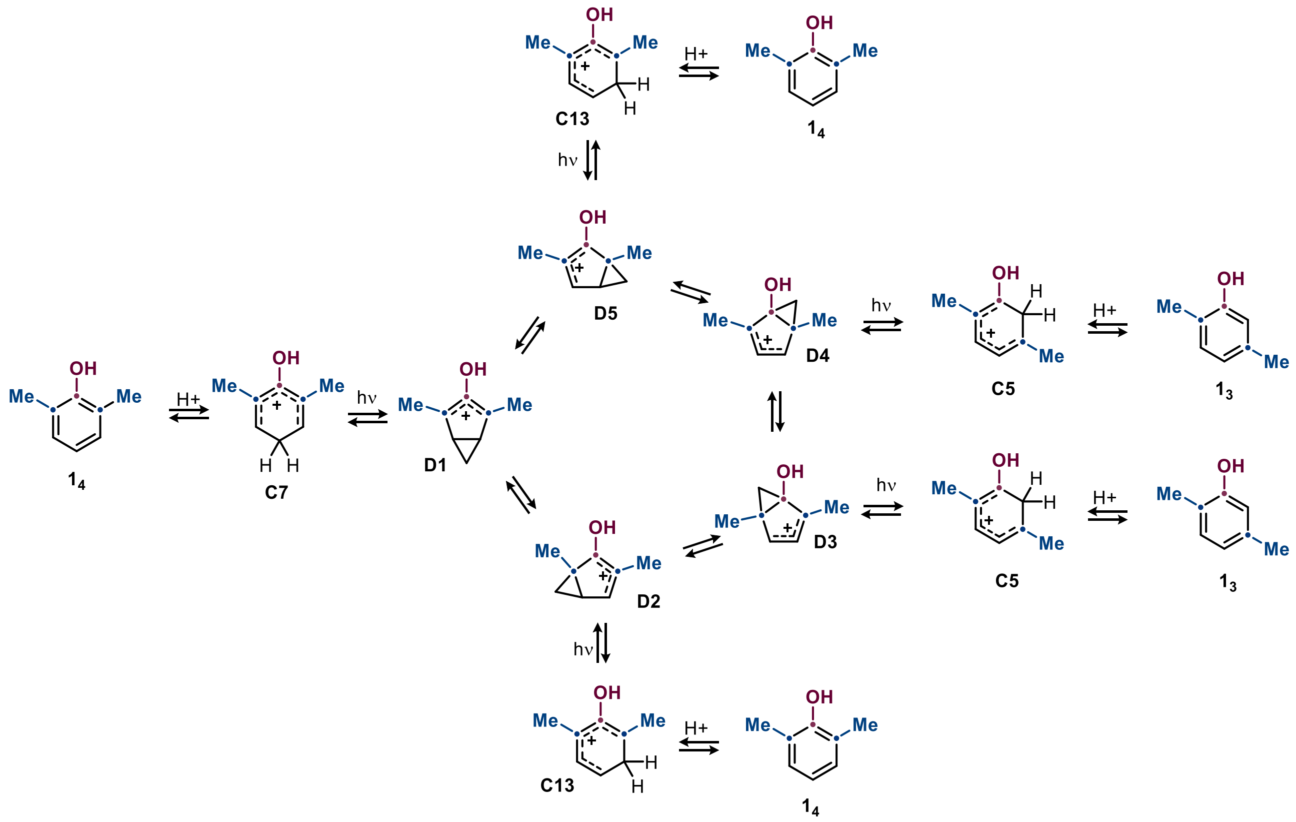

Figure S17. Proposed pathways for the permutation of dimethyl phenols **1**3 and **1**4.

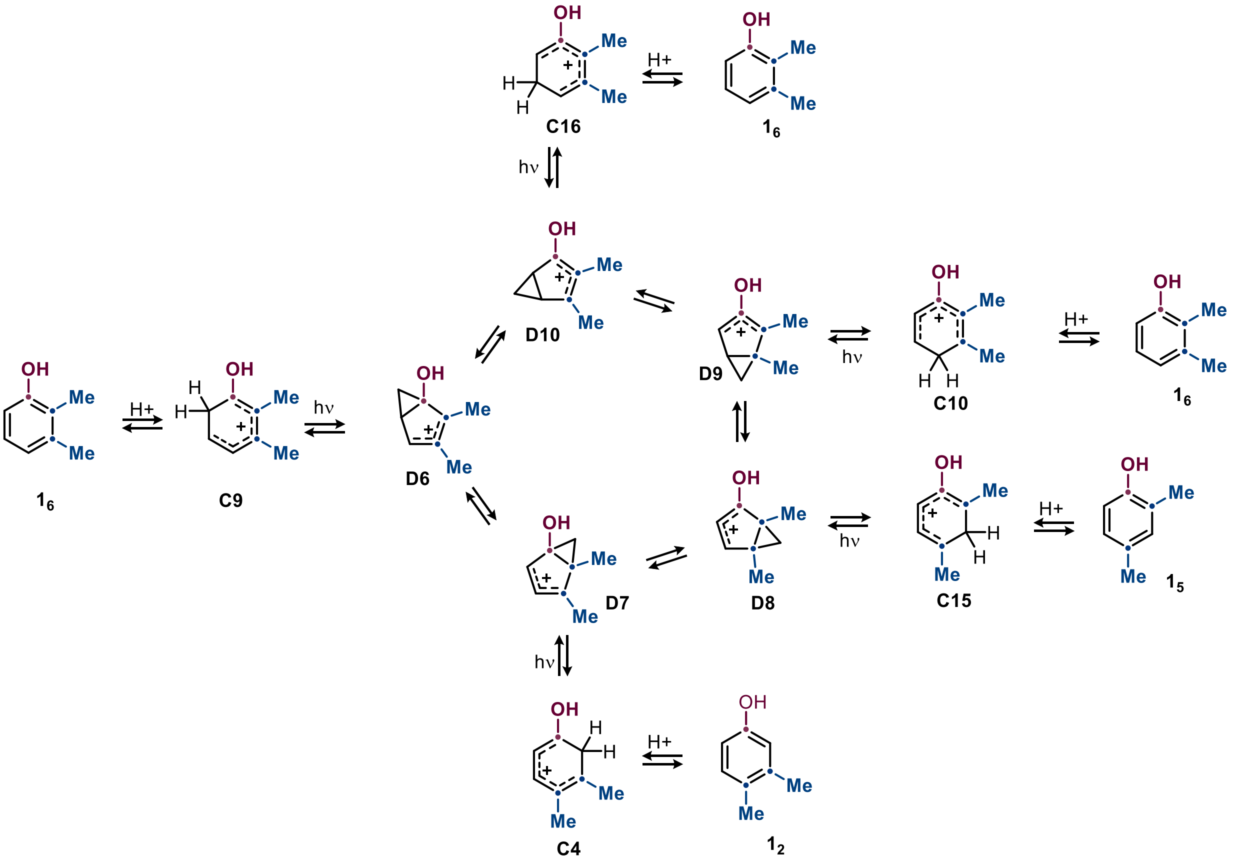

Figure S18. Proposed pathways for the permutation of dimethyl phenols **1_2, 1_5** and **1_6.**

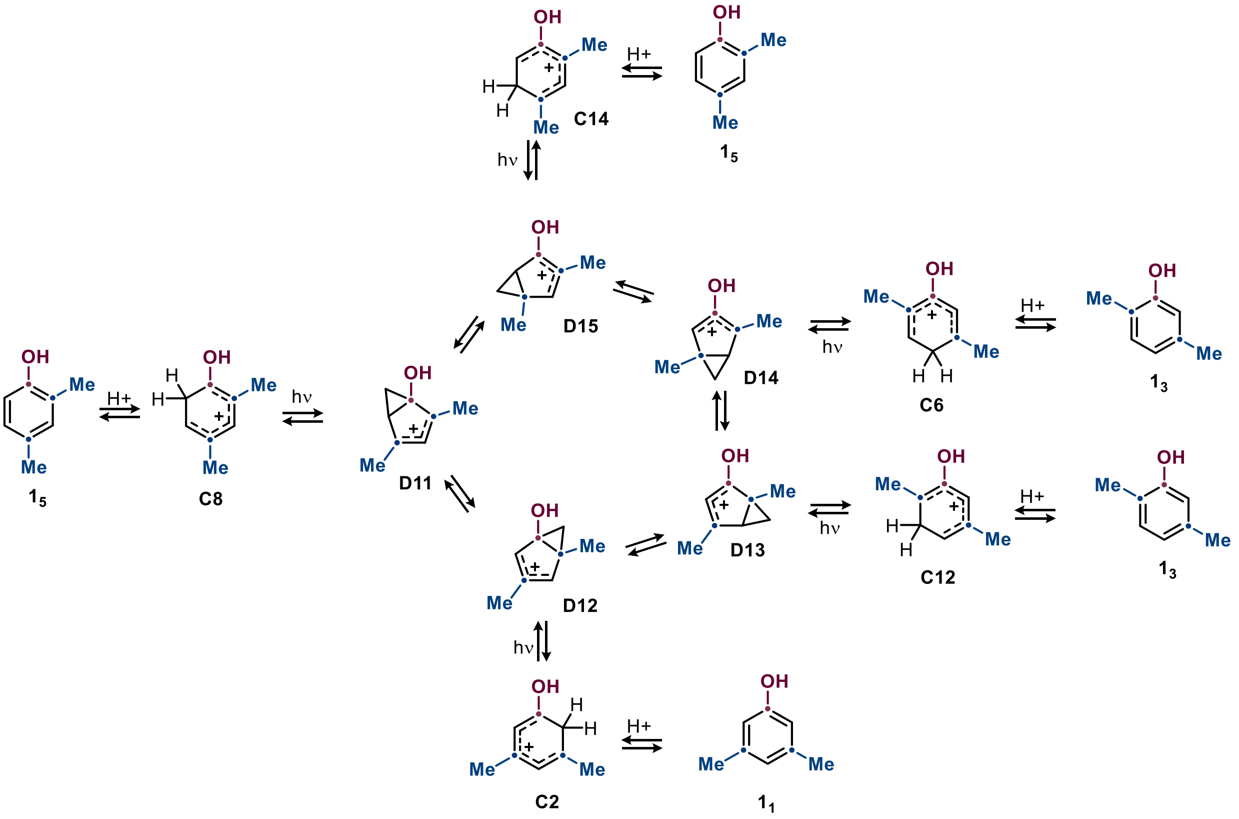

Figure S19. Proposed pathways for the permutation of dimethyl phenols **1_1, 1_3** and **1_5.**

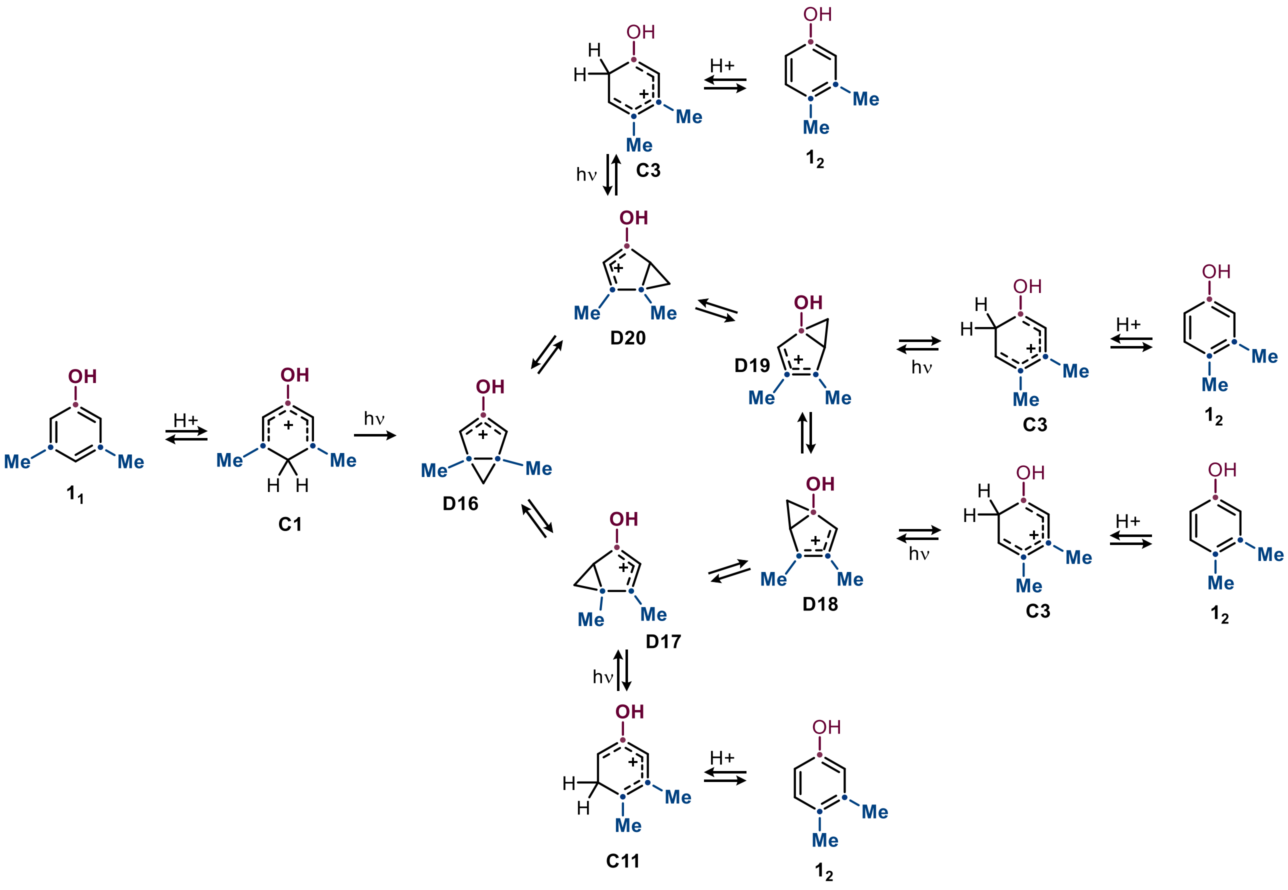

Figure S20. Proposed pathways for the permutation of dimethyl phenols **1_1** and **1_2.**

11 Unsuccessful Substrates

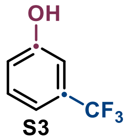

S3

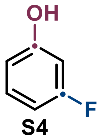

S4

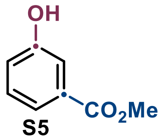

S5

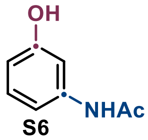

S6

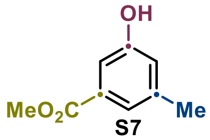

S7

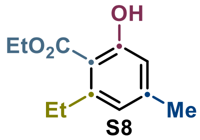

S8

12 NMR Spectra

6₁ – ^{1}H NMR (400 MHz, CDCl$_3$)

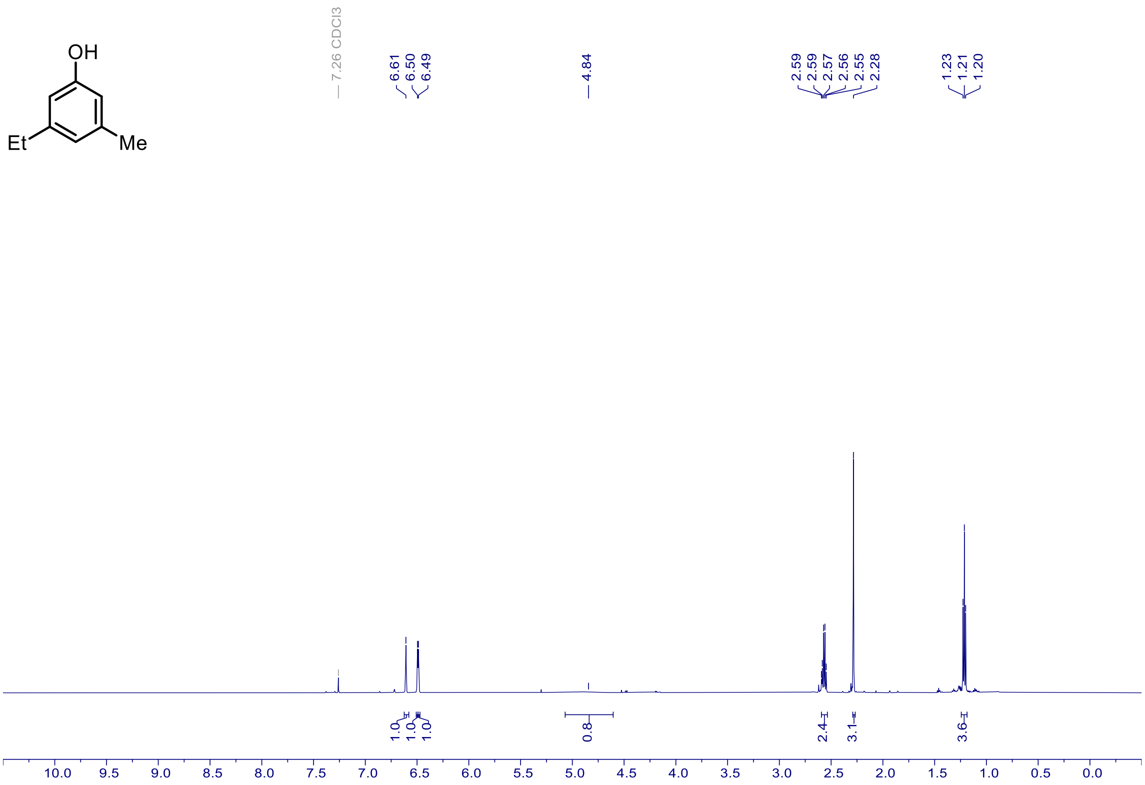

6₁ – ^{13}C NMR (101 MHz, CDCl$_3$)

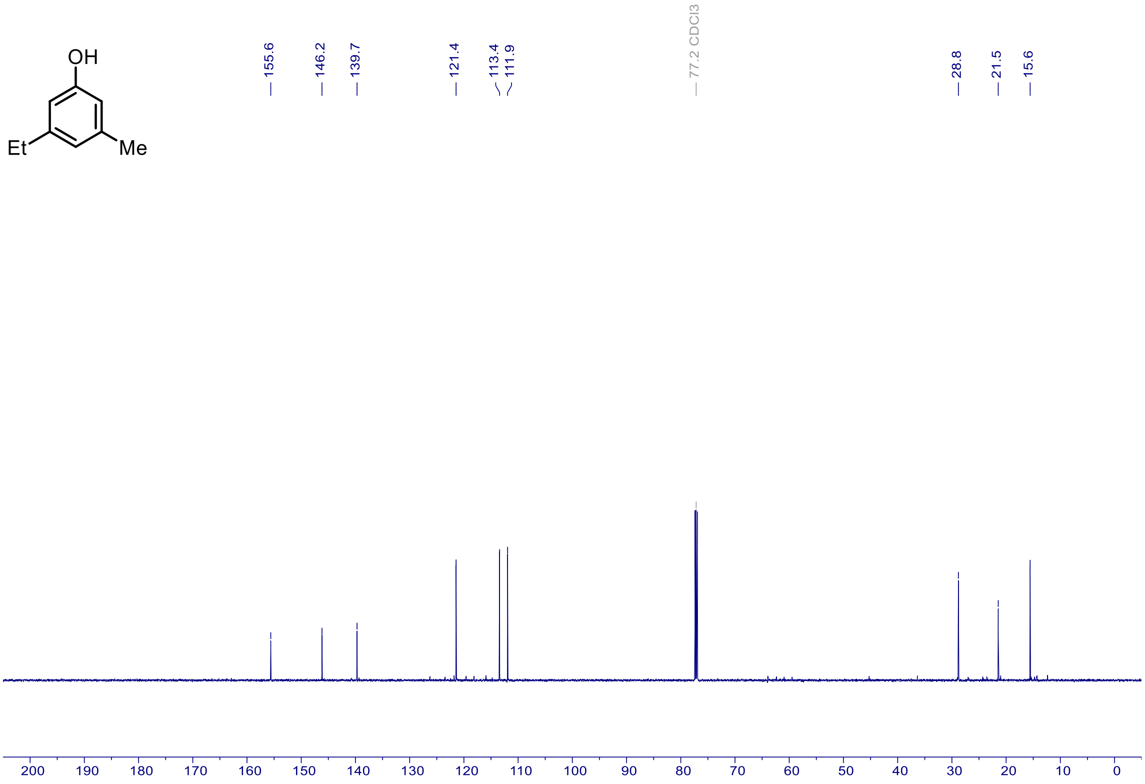

16l – ¹H NMR (400 MHz, $CDCl_3$)

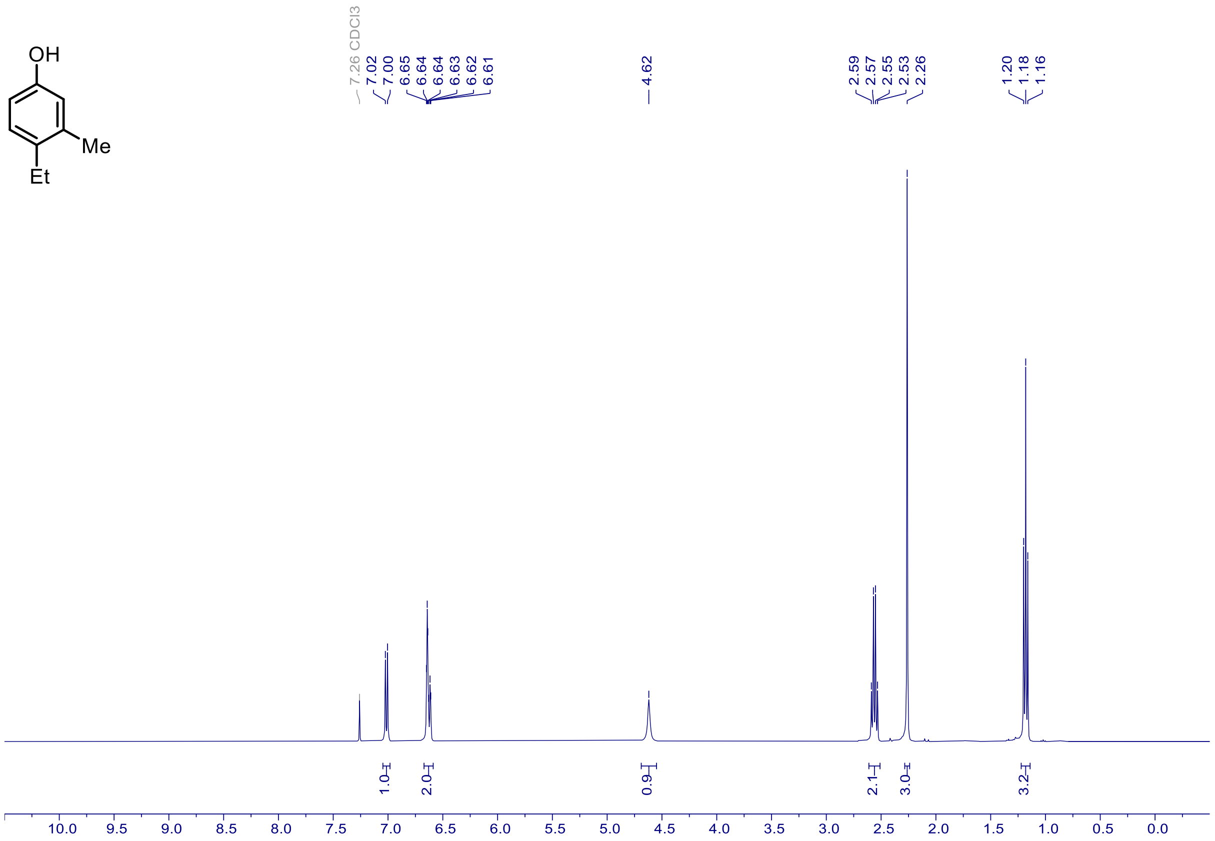

16l – ¹³C NMR (101 MHz, $CDCl_3$)

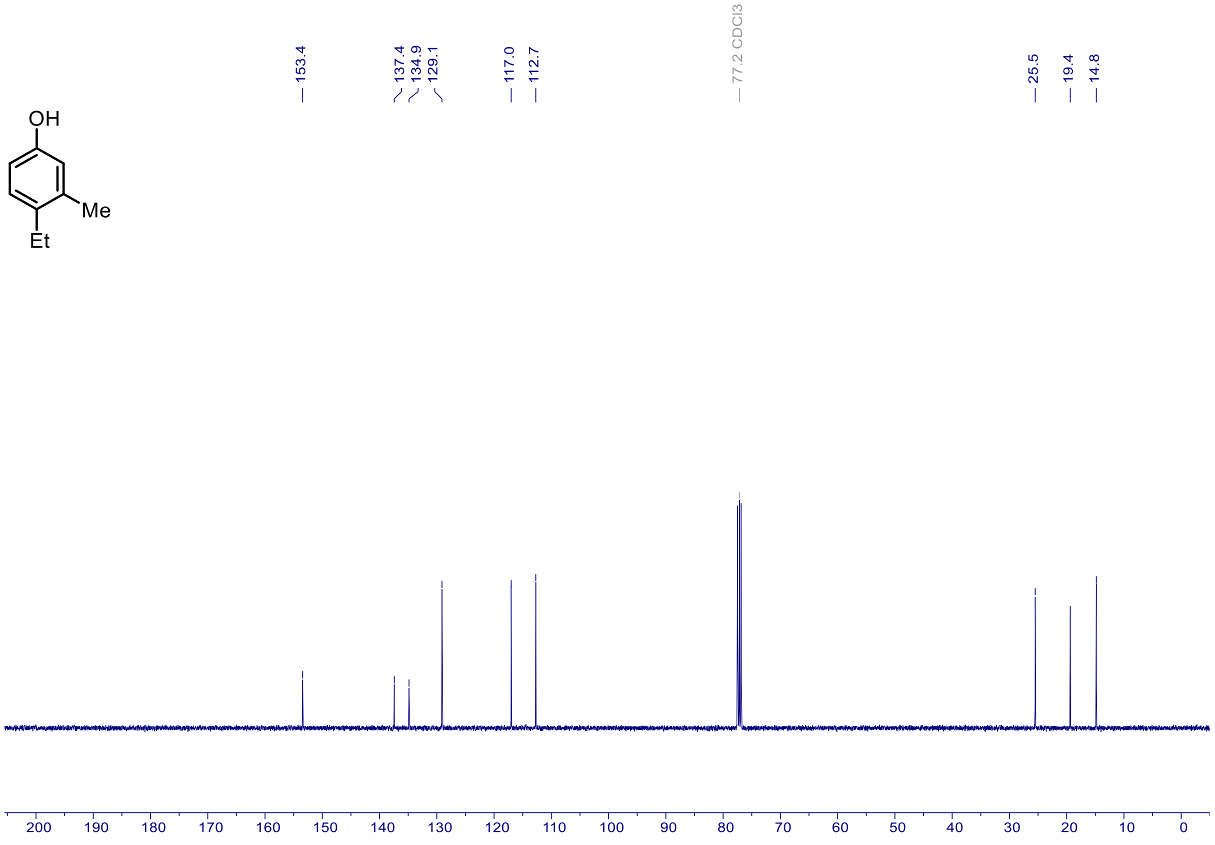

13₃ – ^{1}H NMR (400 MHz, $CDCl_3$)

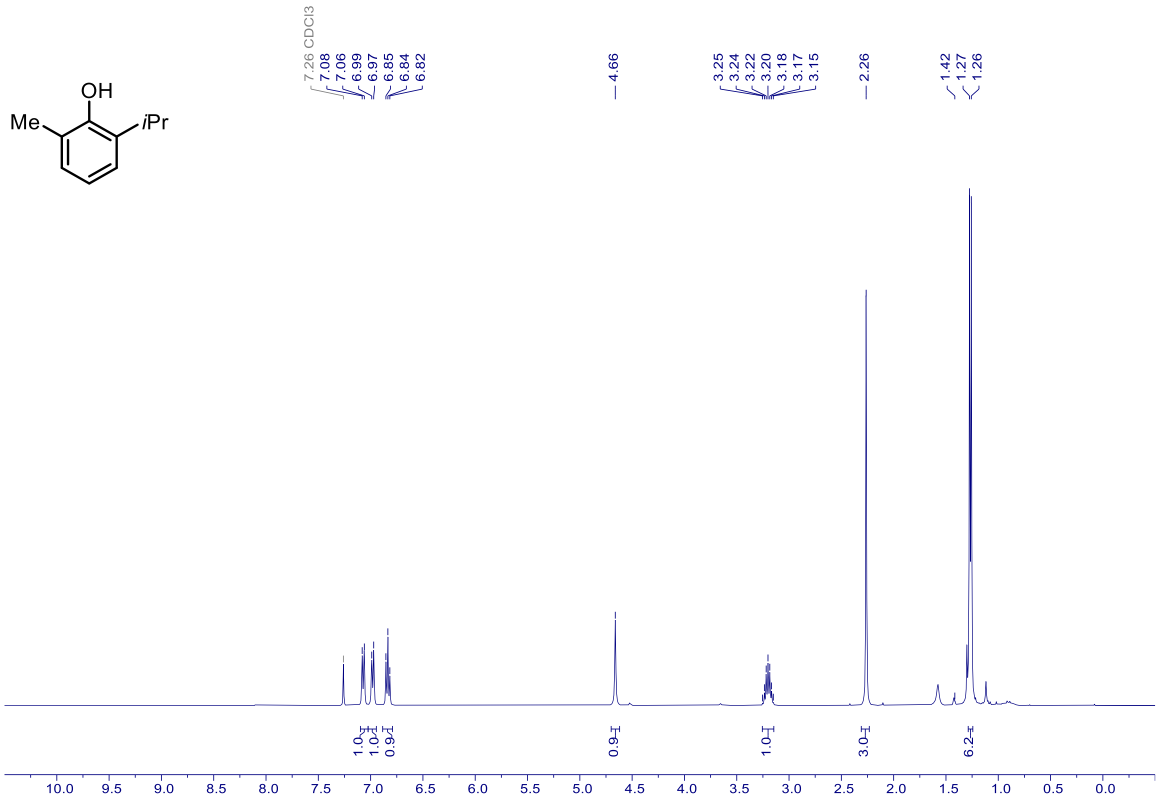

13₃ – ^{13}C NMR (101 MHz, $CDCl_3$)

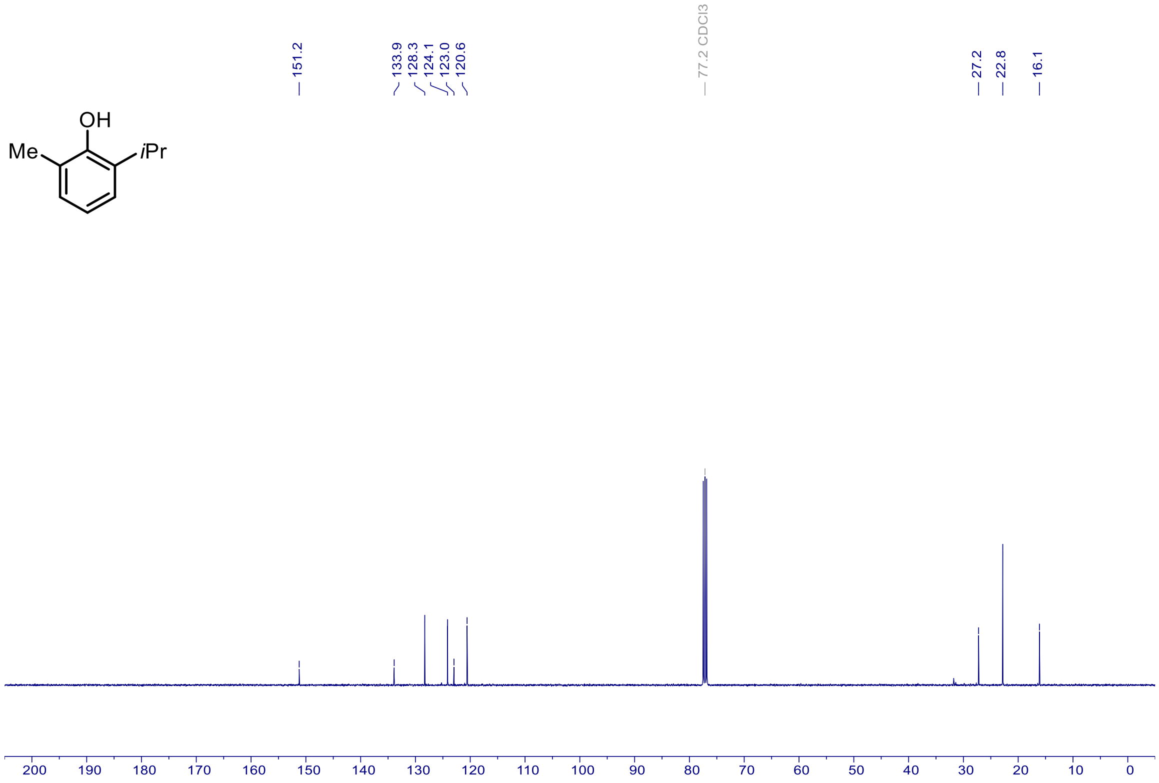

15₂ – ^{1}H NMR (600 MHz, $CDCl_3$)

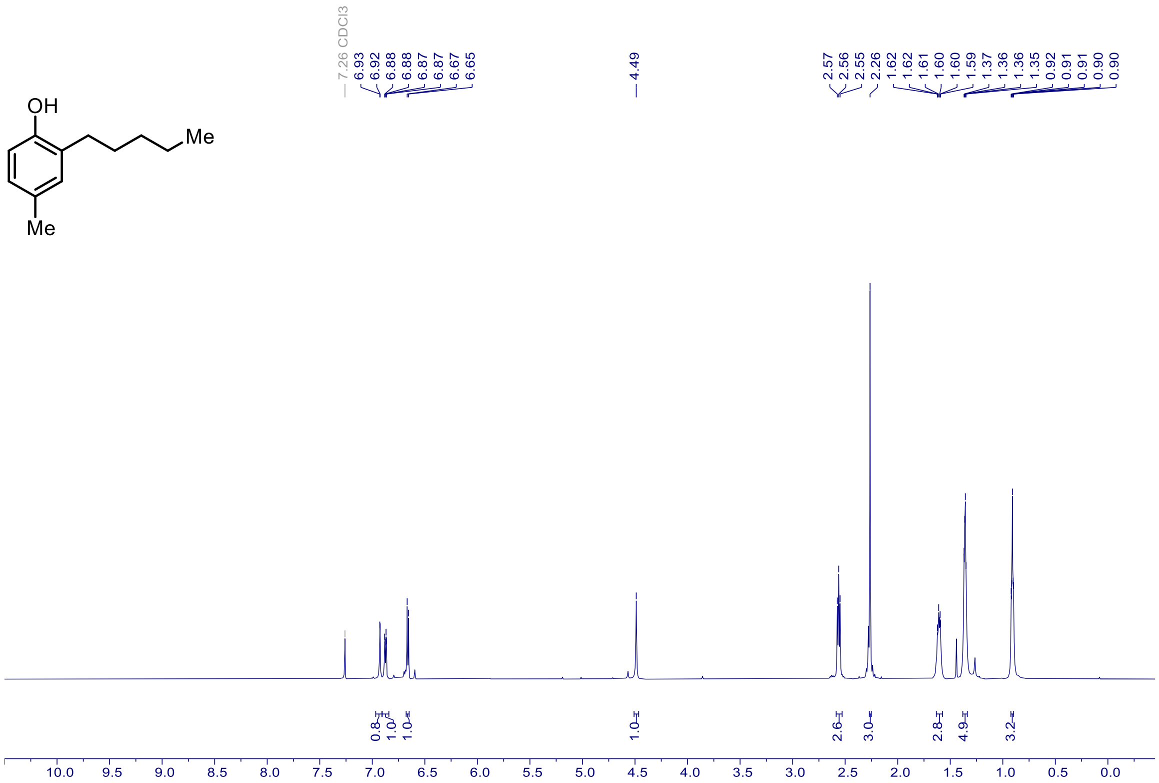

15₂ – ^{13}C NMR (151 MHz, $CDCl_3$)

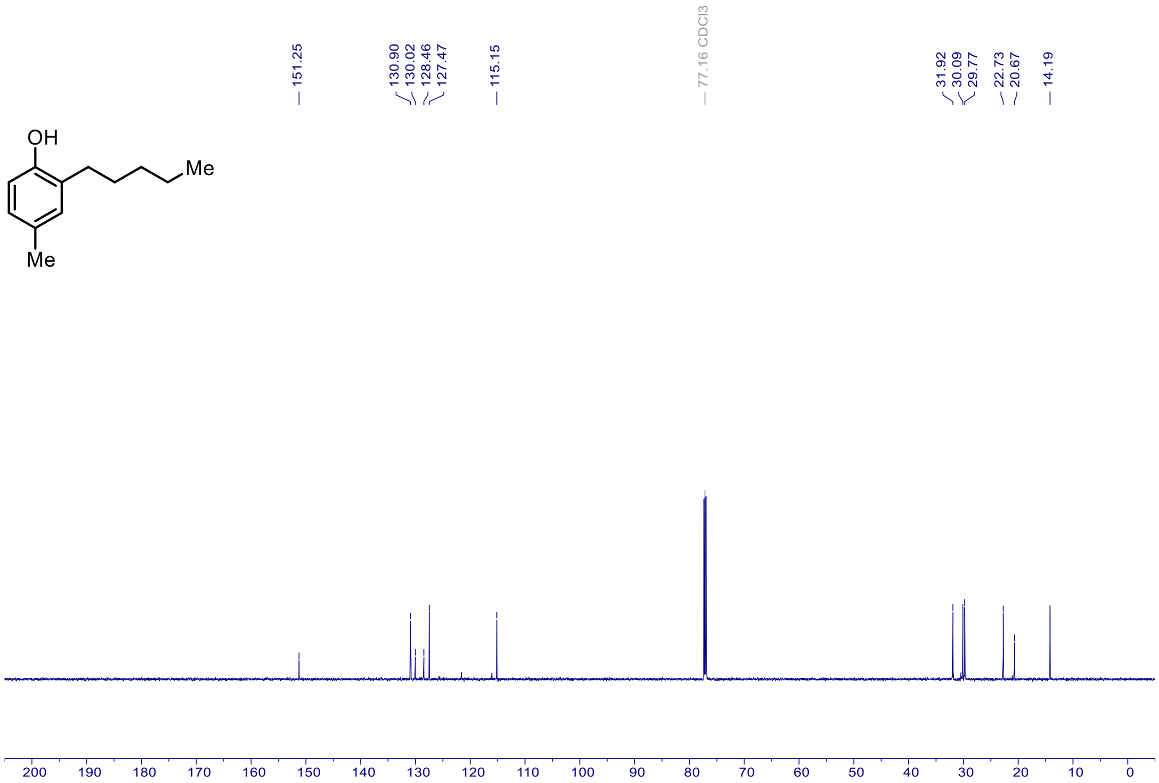

21$_2$ – ^{1}H NMR (600 MHz, $CDCl_3$)

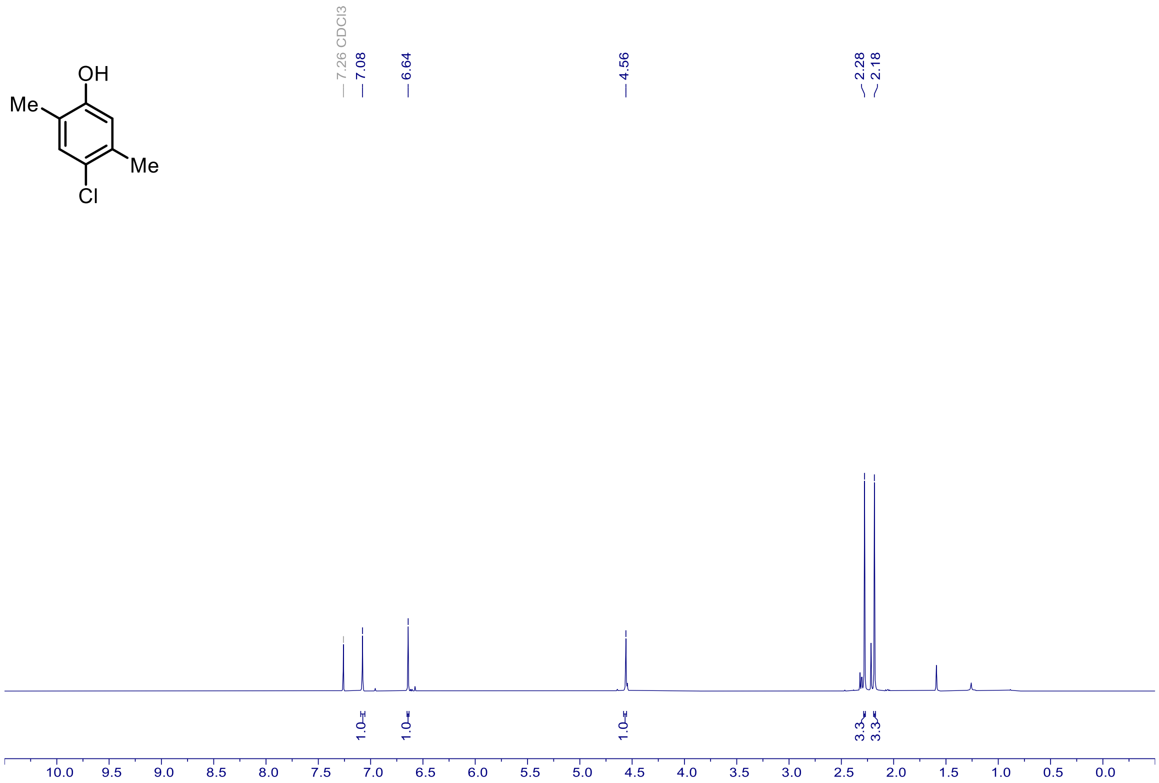

21$_2$ – ^{13}C NMR (151 MHz, $CDCl_3$)

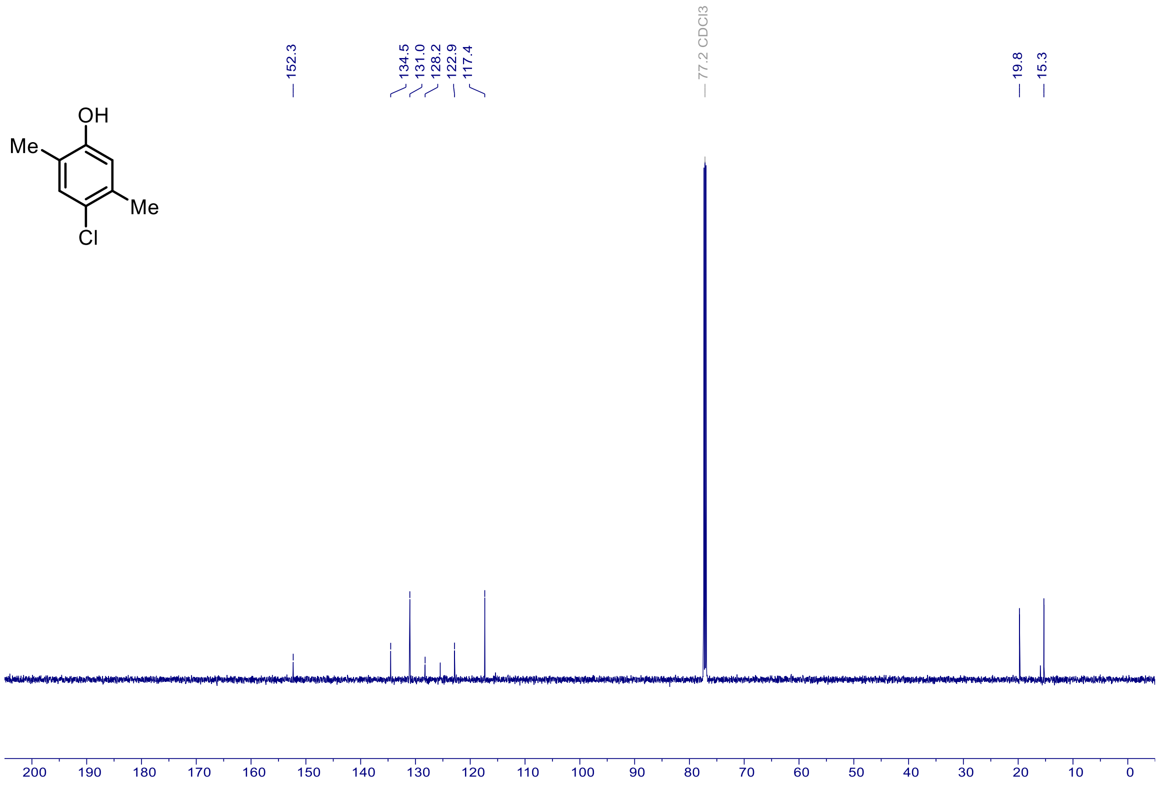

13 Supplementary References

1. Luo, J., Preciado, S., Araromi, S. O. & Larrosa, I. A Domino Oxidation/Arylation/Protodecarboxylation Reaction of Salicylaldehydes: Expanded Access to meta-Arylphenols. *Chemistry – An Asian Journal* **11**, 347–350 (2016).
2. Park, J. H. *et al.* Continuous-Flow Synthesis of meta-Substituted Phenol Derivatives. *Org. Process Res. Dev.* **19**, 812–818 (2015).
3. Yu, G. & Clive, D. L. J. Formation of meta-Substituted Phenols by Transition Metal-Free Aromatization: Use of 2-Bromocyclohex-2-en-1-ones. *J. Org. Chem.* **81**, 8470–8484 (2016).
4. Fischer, A., Henderson, G. N. & Thompson, R. J. Ipso nitration. XIX. Formation of cyclohexadiene adducts from nitration of 4-ethyltoluene and 1,4-diethylbenzene in nitric acid and acetic anhydride. *Aust. J. Chem.* **31**, 1241–1247 (1978).
5. Wu, K., Wu, L.-L., Zhou, C.-Y. & Che, C.-M. Transition-Metal-Free C(sp2)–C(sp2) Cross-Coupling of Diazo Quinones with Catechol Boronic Esters. *Angewandte Chemie International Edition* **59**, 16202–16208 (2020).
6. Ortin, G. G. D. & G. Salles, A. Persulfate-promoted synthesis of biphenyl compounds in water from biomass-derived triacetic acid lactone. *Organic & Biomolecular Chemistry* **20**, 9292–9297 (2022).
7. Krysan, D. J., Gurski, A. & Liebeskind, L. S. A synthesis of highly substituted aromatics through regiocontrolled construction of cyclobutenones bearing unsaturated substituents at the 4-position. *J. Am. Chem. Soc.* **114**, 1412–1418 (1992).
8. Paul, R., Ali, M. A. & Punniyamurthy, T. Copper-Catalyzed Hydroxylation of Aryl Halides with Tetrabutylammonium Hydroxide: Synthesis of Substituted Phenols and Alkyl Aryl Ethers. *Synthesis* **2010**, 4268–4272 (2010).

9. Tu, X.-P., Wei, L.-L., Zhang, K.-X., Chen, Y. & Zhou, M.-D. Synthesis of fluorescein-containing polymeric heterogeneous photocatalyst and its applications. *Tetrahedron* **160**, 134028 (2024).
10. Duff, L. *et al.* Denitrative Hydroxylation of Unactivated Nitroarenes. *Chemistry – A European Journal* **29**, e202203807 (2023).
11. Zhao, Y., Yu, C., Liang, W., L. Atodiresei, I. & W. Patureau, F. TEMPO-mediated late stage photochemical hydroxylation of biaryl sulfonium salts. *Chemical Communications* **58**, 2846–2849 (2022).
12. Zhao, X., Bai, L., Li, J. & Jiang, X. Photouranium-Catalyzed C–F Activation Hydroxylation via Water Splitting. *J. Am. Chem. Soc.* **146**, 11173–11180 (2024).
13. Chan, C.-C., Chen, Y.-W., Su, C.-S., Lin, H.-P. & Lee, C.-F. Green Catalysts Derived from Agricultural and Industrial Waste Products: The Preparation of Phenols from CsOH and Aryl Iodides using CuO on Mesoporous Silica. *European Journal of Organic Chemistry* **2011**, 7288–7293 (2011).
14. Cuadros, S. *et al.* The Photochemical Activity of a Halogen-Bonded Complex Enables the Microfluidic Light-Driven Alkylation of Phenols. *Org. Lett.* **24**, 2961–2966 (2022).
15. Lavery, C. B., Rotta-Loria, N. L., McDonald, R. & Stradiotto, M. Pd2dba3/Bippyphos: A Robust Catalyst System for the Hydroxylation of Aryl Halides with Broad Substrate Scope. *Advanced Synthesis & Catalysis* **355**, 981–987 (2013).
16. Lee, D.-H., Kwon, K.-H. & Yi, C. S. Dehydrative C–H Alkylation and Alkenylation of Phenols with Alcohols: Expedient Synthesis for Substituted Phenols and Benzofurans. *J. Am. Chem. Soc.* **134**, 7325–7328 (2012).
17. Fischer, A. & Henderson, G. N. ipso Nitration. XXIII. Reactions of cyclohexadiene adducts from nitration of 4-ethyltoluene in acetic anhydride. *Can. J. Chem.* **59**, 2314–2327 (1981).

18. Cazorla, C., De Vries, T. S. & Vedejs, E. P-Directed Borylation of Phenols. *Org. Lett.* **15**, 984–987 (2013).
19. Pontillo, J., Gao, Y., Wade, W. S., Wu, D. & Eccles, W. K. Monoamine Re-Uptake Inhibitors and Methods Relating Thereto. (2006).
20. Samkian, A. E., Sercel, Z. P., Virgil, S. C. & Stoltz, B. M. Some unusual transformations of a highly reactive α-bromocaranone. *Tetrahedron Letters* **89**, 153496 (2022).
21. Martin, R. & Demerseman, P. Lewis acids catalysed Fries rearrangement of isopropylcresol esters. *Monatsh Chem* **121**, 227–236 (1990).
22. Katritzky, A. R., Lan, X. & Lam, J. N. o-(α-Benzotriazolylalkyl)phenols: Versatile Intermediates for the Synthesis of Substituted Phenols. *Chemische Berichte* **124**, 1809–1817 (1991).
23. Goclik, L., Offner-Marko, L., Bordet, A. & Leitner, W. Selective hydrodeoxygenation of hydroxyacetophenones to ethyl-substituted phenol derivatives using a FeRu@SILP catalyst. *Chem. Commun.* **56**, 9509–9512 (2020).
24. Chen, G. *et al.* Demethylaromatization of cyclohexadienones by iodotriphenylphosphonium iodide. *Tetrahedron Letters* **117**, 154365 (2023).
25. Rao, M. L. N. & Meka, S. Pd-catalyzed protecting-group-free cross-couplings of iodophenols with atom-economic triarylbismuth reagents. *Tetrahedron Letters* **61**, 151512 (2020).
26. Selt, M., Mentizi, S., Schollmeyer, D., Franke, R. & Waldvogel, S. R. Selective and Scalable Dehydrogenative Electrochemical Synthesis of 3,3′,5,5′-Tetramethyl-2,2′-biphenol. *Synlett* **30**, 2062–2067 (2019).
27. Xia, Q. *et al.* Solvent-switchable regioselective 1,2- or 1,6-addition of quinones with boronic acids. *Chemical Communications* **59**, 8416–8419 (2023).

28. Zhu, J., Xue, Y., Zhang, R., Ratchford, B. L. & Dong, G. Catalytic Activation of Unstrained C(Aryl)–C(Alkyl) Bonds in 2,2′-Methylenediphenols. *J. Am. Chem. Soc.* **144**, 3242–3249 (2022).

29. Kutchin, A. V., Fedorova, I. V., Loginova, I. V. & Chukicheva, I. Yu. Features of the use of ClO2 in the oxidation of some alkylphenols. *Russ Chem Bull* **72**, 202–212 (2023).

30. Rinkel, J., Babczyk, A., Wang, T., Stadler, M. & Dickschat, J. S. Volatiles from the hypoxylaceous fungi Hypoxylon griseobrunneum and Hypoxylon macrocarpum. *Beilstein J. Org. Chem.* **14**, 2974–2990 (2018).

31. Masarwa, A., Weber, M. & Sarpong, R. Selective C–C and C–H Bond Activation/Cleavage of Pinene Derivatives: Synthesis of Enantiopure Cyclohexenone Scaffolds and Mechanistic Insights. *J. Am. Chem. Soc.* **137**, 6327–6334 (2015).

32. Castro-Godoy, W. D. *et al.* Linker-Assisted CdS-TiO2 Nanohybrids as Reusable Visible Light Photocatalysts for the Oxidative Hydroxylation of Arylboronic Acids. *J. Org. Chem.* **88**, 6489–6497 (2023).

33. Zhu, M.-H. *et al.* Detosylative (Deutero)alkylation of Indoles and Phenols with (Deutero)alkoxides. *Org. Lett.* **21**, 7073–7077 (2019).

34. Wu, Z., Wei, F., Wan, B. & Zhang, Y. Pd-Catalyzed ipso,meta-Dimethylation of ortho-Substituted Iodoarenes via a Base-Controlled C–H Activation Cascade with Dimethyl Carbonate as the Methyl Source. *J. Am. Chem. Soc.* **143**, 4524–4530 (2021).

35. Levin, N. *et al.* Decarboxylation and Tandem Reduction/Decarboxylation Pathways to Substituted Phenols from Aromatic Carboxylic Acids Using Bimetallic Nanoparticles on Supported Ionic Liquid Phases as Multifunctional Catalysts. *J. Am. Chem. Soc.* **145**, 22845–22854 (2023).

36. Lu, H. *et al.* Aerobic Oxidative Hydroxylation of Arylboronic Acids under Visible-Light Irradiation without Metal Catalysts or Additives. *Org. Lett.* **26**, 1959–1964 (2024).

37. Yanai, T., Tew, D. P. & Handy, N. C. A new hybrid exchange–correlation functional using the Coulomb-attenuating method (CAM-B3LYP). *Chemical Physics Letters* **393**, 51–57 (2004).

38. Dunning, T. H., Jr. Gaussian basis sets for use in correlated molecular calculations. I. The atoms boron through neon and hydrogen. *The Journal of Chemical Physics* **90**, 1007–1023 (1989).

39. Marenich, A. V., Cramer, C. J. & Truhlar, D. G. Universal Solvation Model Based on Solute Electron Density and on a Continuum Model of the Solvent Defined by the Bulk Dielectric Constant and Atomic Surface Tensions. *J. Phys. Chem. B* **113**, 6378–6396 (2009).

40. Casida, M. E. & Huix-Rotllant, M. Progress in Time-Dependent Density-Functional Theory. *Annual Review of Physical Chemistry* **63**, 287–323 (2012).

41. Neese, F. The ORCA program system. *WIREs Computational Molecular Science* **2**, 73–78 (2012).

42. Furche, F. *et al.* Turbomole. *WIREs Computational Molecular Science* **4**, 91–100 (2014).

43. Gaussian 16, Revision C.01, M. J. Frisch, G. W. Trucks, H. B. Schlegel, G. E. Scuseria, M. A. Robb, J. R. Cheeseman, G. Scalmani, V. Barone, G. A. Petersson, H. Nakatsuji, X. Li, M. Caricato, A. V. Marenich, J. Bloino, B. G. Janesko, R. Gomperts, B. Mennucci, H. P. Hratchian, J. V. Ortiz, A. F. Izmaylov, J. L. Sonnenberg, D. Williams-Young, F. Ding, F. Lipparini, F. Egidi, J. Goings, B. Peng, A. Petrone, T. Henderson, D. Ranasinghe, V. G. Zakrzewski, J. Gao, N. Rega, G. Zheng, W. Liang, M. Hada, M. Ehara, K. Toyota, R. Fukuda, J. Hasegawa, M. Ishida, T. Nakajima, Y. Honda, O. Kitao, H. Nakai, T. Vreven, K. Throssell, J. A. Montgomery, Jr., J. E. Peralta, F. Ogliaro, M. J. Bearpark, J. J. Heyd, E. N. Brothers, K. N. Kudin, V. N. Staroverov, T. A. Keith, R. Kobayashi, J. Normand, K. Raghavachari, A. P. Rendell, J. C. Burant, S. S. Iyengar, J. Tomasi, M. Cossi, J. M. Millam, M. Klene, C. Adamo, R. Cammi, J. W. Ochterski, R. L. Martin, K. Morokuma, O. Farkas, J. B. Foresman, and D. J. Fox, Gaussian, Inc., Wallingford CT (2016).
